# Internal feedback circuits among MEX-5, MEX-6, and PLK-1 maintain faithful patterning in the *Caenorhabditis elegans* embryo

**DOI:** 10.1073/pnas.2407517121

**Published:** 2024-12-17

**Authors:** Alexandre Pierre Vaudano, Françoise Schwager, Monica Gotta, Sofia Barbieri

**Affiliations:** ^a^Department of Cell Physiology and Metabolism, Faculty of Medicine, University of Geneva, 1211 Geneva, Switzerland

**Keywords:** reaction–diffusion, RNA binding proteins, MEX-5, MEX-6, PLK-1, polarity feedback loops, Monte Carlo simulations

## Abstract

Proteins become asymmetrically distributed in the one-cell *Caenorhabditis elegans* embryo thanks to reaction–diffusion mechanisms that are often entangled in complex feedback loops. Cortical polarity drives the enrichment of the RNA-binding proteins MEX-5 and MEX-6 in the anterior cytoplasm through concentration gradients. MEX-5 and MEX-6 promote the patterning of other cytoplasmic factors, including that of the anteriorly enriched mitotic polo-like kinase PLK-1, but also contribute to proper cortical polarity. The gradient of MEX-5 forms through a differential-diffusion mechanism. How MEX-6 establishes a gradient and how MEX-5 and MEX-6 regulate cortical polarity is not known. Here, we reveal that the two MEX proteins develop concentration asymmetries via similar mechanisms, but despite their strong sequence homology, they differ in terms of how their concentration gradients are regulated. We find that PLK-1 promotes the enrichment of MEX-5 and MEX-6 at the anterior through different circuits: PLK-1 influences the MEX-5 gradient indirectly by regulating cortical polarity while it modulates the formation of the gradient of MEX-6 through its physical interaction with the protein. We thus propose a model in which PLK-1 mediates protein circuitries between MEX-5, MEX-6, and cortical proteins to faithfully establish and maintain polarity.

## Significance

Proteins get asymmetrically enriched in the one-cell *Caenorhabditis elegans* embryo thanks to reaction–diffusion mechanisms that often rely on complex feedback loops. Starting from the investigation of the biophysical mechanisms of MEX-6 gradient formation, we here unveil a complex interplay among the cytoplasmic RNA-binding proteins MEX-5 and MEX-6 and the polo-like kinase PLK-1. Despite the strong homology between MEX-5 and MEX-6, we suggest that their anterior-rich gradients are regulated by PLK-1 through two different feedback circuits: PLK-1 influences the gradient of MEX-5 by regulating cortical polarity, whereas it modulates the formation of MEX-6 gradient by physically interacting with it.

Molecular patterning is a fascinating mechanism that challenges the rules of physics. First, establishing asymmetries requires to counteract the innate molecule tendency to randomly distribute due to Brownian diffusion. Second, defining precise asymmetries requires multiple mechanisms, which often feedback into each other in order to guarantee robustness against perturbations or failures. Third, once a steady-state configuration is reached, the pattern must be maintained, e.g., for the duration of a cell division, making polarity establishment and maintenance a very dynamic process. This is even more emphasized when rescaling this process at the micrometer level, like in the intracellular context. A well-described mechanism to create patterning within individual cells is via the regulation of the molecular properties by means of biochemical reactions, through the so-called reaction–diffusion mechanisms. Subcellular concentration gradients were shown to be achieved by modulating the diffusivity of proteins in a space-dependent manner (differential-diffusion mechanism) (1, 2).

Reaction–diffusion mechanisms underlie the dynamics of several polarity factors in the one-cell *Caenorhabditis elegans* (*C. elegans*) embryo, starting with the PARtitioning defective (PAR) proteins. Two classes of PAR proteins, the anterior and posterior PAR proteins (referred to as aPARs and pPARs), associate with the membrane (3–7) and become segregated to the anterior and posterior domains by advection by the actomyosin flows (8–17). Kinase-dependent mutual antagonism helps aPAR and pPAR to exclude each other from the cortex and create mirroring gradients at the equilibrium (10, 12, 18–20). This molecular patterning at the cortex then signals to the cytoplasm, where proteins differentially segregate along the anteroposterior axis (21–27). Examples of anteriorly enriched proteins are the RNA-binding proteins MEX-5 and MEX-6 (Muscle-EXcess proteins) and the mitotic Polo-Like Kinase 1 (PLK-1) (11, 21, 22, 26, 28–32).

**Fig. 1.**
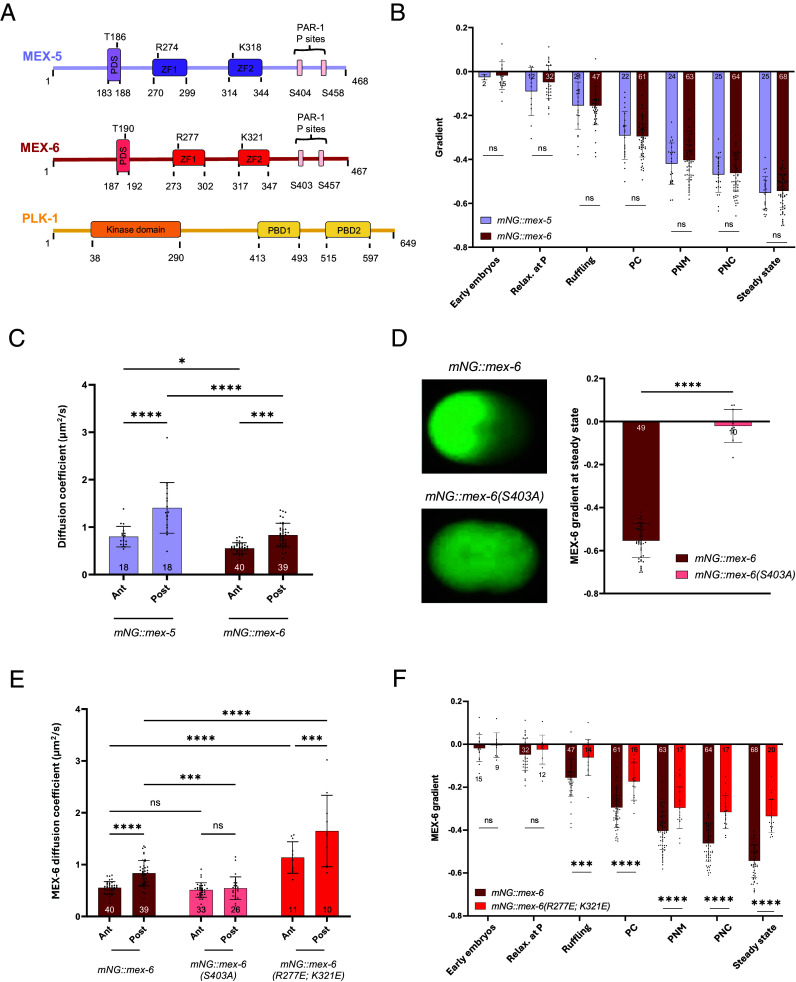
MEX-6 forms a gradient through a reaction–diffusion mechanism but diffuses slower than MEX-5. (*A*) Scheme of the MEX-5, MEX-6, and PLK-1 protein structures, showing the main interaction domains and the amino acids mutagenized in vivo by CRISPR. (*B*) mNG::MEX-5 and mNG::MEX-6 gradients as a function of time, shown for relevant timepoints during the first cell division (see legend below). The gradient is quantified as the slope of the linear fit of the signal along the anterior–posterior axis (∂Intensity/∂x). For simplicity, it will be referred to as “Gradient” or “Protein gradient” (e.g., MEX-6 gradient) in all the subsequent graphs. (*C*) Comparison of mNG::MEX-5 and mNG::MEX-6 diffusivity at both the anterior and posterior poles of one-cell embryos, at steady state. (*D*) *Left*, images of one-cell embryos showing the steady-state localization of mNG::MEX-6 and mNG::MEX-6(S403A); *Right*, quantification of their gradients at steady state. (*E*) Quantification of MEX-6 diffusion coefficient in the parental *mNG::mex-6* strain and in the *mNG::mex-6(S403A)* and *mNG::mex-6(R277E; K321E)* mutants, measured at the anterior and posterior poles of embryos at steady state. (*F*) Comparison of mNG::MEX-6 and mNG::MEX-6(R277E; K321E) gradients as a function time. In (*B*–*F*), the bars represent the average values of different measurements, and the error bars the SD. The number N of analyzed embryos is reported for each condition. Embryos from different experiments were pooled together and, for (*B*) and (*C*), these bar plots were used as control conditions in the following experiments. In (*B*, *D*, and *F*), the statistical analysis was performed, for each stage separately, using the two-tailed unpaired *t* test. In (*C*) and (*E*), the statistical analysis was done using the two-way ANOVA test, with Tukey’s multiple comparison. Legend: Relax. at P = relaxation at posterior; PC = pseudocleavage; PNM = pronuclear meeting; PNC = pronuclear centration. Ant = anterior; Post = posterior. For the statistics: ns: *P* > 0.5, *P < 0.05, **P < 0.01, ***P < 0.001, ****P < 0.0001. These conventions are kept in all figures.

MEX-5 and MEX-6 share ∼70% protein identity and 85% similarity and contain a polo-docking site (PDS), where PLK-1 can bind (30), two CCCH-rich zinc finger (ZF) domains, responsible for the loading of RNAs (26, 33–35), and two PAR-1 phosphorylation sites at the C terminus (Fig. 1*A*). *mex-5* mutant embryos are nonviable, while MEX-6 depletion or mutation does not reduce viability. However, depletion or mutation of MEX-6 in a *mex-5* background gives stronger early embryonic phenotypes, indicating that MEX-6 plays a redundant role with MEX-5 in the early embryo (28).

MEX-5 and MEX-6 interact with PLK-1 in yeast two-hybrid assays (30), and this interaction mediates the formation of an anterior-rich PLK-1 gradient. Depletion of MEX-5 or MEX-6 partially reduces the PLK-1 gradient (23, 25, 30–32, 36), and the depletion of both MEX proteins results in a uniformly distributed kinase (23, 30). Depletion of both MEX-5 and MEX-6 also leads to defects in cortical polarity (3, 11, 28) in a yet unknown mechanism, indicating that a feedback regulation between cortical and cytoplasmic polarity exists.

The enrichment of MEX-5 at the anterior is regulated by a reaction–diffusion mechanism that depends on the posterior kinase PAR-1 (pPAR), and on a uniformly localized PP2A phosphatase. PAR-1 phosphorylates MEX-5 and prevents binding to RNA complexes, turning the slow-diffusing MEX-5 molecules into a fast-diffusing state that equalizes its distribution throughout the cytoplasm (26). As PAR-1 activity is higher at the posterior (37), an imbalance between phosphatase and kinase activity is created at the anterior, where the dephosphorylated component of MEX-5 slows down by forming complexes with RNAs and becomes enriched (26). How MEX-6 establishes its anterior gradient remains uncharacterized.

Here, we investigate the formation of the MEX-6 gradient, which we show to happen by a reaction–diffusion mechanism similar to that of MEX-5. However, despite the high sequence homology between the two proteins, MEX-6 displays a lower diffusion coefficient than MEX-5 and their gradients are regulated in a different manner. We find that MEX-5 and MEX-6 influence each other’s gradient. We furthermore reveal that PLK-1 regulates the establishment and maintenance of MEX-5 and MEX-6 gradients through distinct feedback circuits involving different polarity factors: on the one hand, PLK-1 regulates the establishment of the MEX-5 gradient indirectly by influencing cortical PAR polarity. On the other hand, PLK-1 modulates the MEX-6 gradient via its physical interaction with MEX-6. Since PLK-1 gradient formation depends on MEX-5 and MEX-6, our data reveal a complex cross talk between these proteins and suggest that one mechanism by which the feedback between cortical and cytoplasmic polarity occurs is via PLK-1.

## Results

### MEX-6 Diffuses Slower than MEX-5.

MEX-5 has a redundant homologue, MEX-6 (Fig. 1*A*), which also forms an anterior-rich cytoplasmic gradient in the one-cell embryo (Movie S1*A*) (11, 28). We examined whether MEX-6 gradient follows the same dynamics as the MEX-5 gradient, using a strain where the endogenous *mex-6* was fused to mNeonGreen by CRISPR. We quantified the gradient of the distribution of mNG::MEX-6 intensity in early one-cell embryos and observed that it became steeper over time (Fig. 1*B* and Movie S1*A*), reaching the maximum absolute value of 0.55 ± 0.08 at steady state (defined as the stages after nuclear envelope break-down). The dynamics and the absolute values of the gradient resembled the ones observed for MEX-5 in the *mNG::mex-5* strain (Fig. 1*B* and Movie S1*B*). The gradient formation followed similar dynamics in strains where the two MEX proteins were tagged with GFP (*SI Appendix*, Fig. S1*A*).

The gradient of MEX-5 is driven by differential diffusion (21, 22, 26). We first asked whether the average diffusivity of MEX-6 also differed from anterior to posterior. We used fluorescence recovery after photobleaching (FRAP) microscopy to measure MEX-6 diffusivity at the steady state and found that it displayed a lower value at the anterior compared to the posterior in both the *mNG::mex-6* strain (0.55 ± 0.12 vs. 0.83 ± 0.25 µm^2^/s, Fig. 1*C* and *SI Appendix*, Table S3) and the *GFP::mex-6* strain (0.49 ± 0.12 vs. 1.00 ± 0.48 µm^2^/s, *SI Appendix*, Fig. S1*B* and Table S3). This trend was in agreement with the published literature for the MEX-5 reaction–diffusion mechanism (21, 22, 26). However, MEX-6 displayed lower absolute values of diffusivity at both the anterior and the posterior compared to those of MEX-5 (Fig. 1*C* and *SI Appendix*, Fig. S1*B*), indicating that MEX-6 diffused slower in the cytoplasm.

Differential diffusion of MEX-5 depends on PAR-1-dependent phosphorylation, converting MEX-5 to a fast species predominantly at the posterior, and on dephosphorylation by a PP2A phosphatase, that transforms fast MEX-5 to a slow species that is hindered in its motion by the formation of complexes with RNAs (26). The sites on MEX-5 that are phosphorylated by PAR-1 (S404 and S458) are conserved in MEX-6 (S403 and S457, Fig. 1*A*) (26). To address whether phosphorylation of MEX-6 is required for its gradient formation, we mutagenized MEX-6 at the conserved site S403 to a nonphosphorylatable form [*mex-6(S403A)*]. We measured the gradient of MEX-6(S403A) at the steady state and found that this mutation alone prevented the formation of a gradient in both the *mNG::mex-6(S403A)* and the *GFP::mex-6(S403A)* strains (Fig. 1*D* and *SI Appendix*, Fig. S1*C*). This suggests that the formation of the MEX-6 gradient happens by a mechanism similar to that involved in the formation of the MEX-5 gradient. To investigate whether the mutation of this amino acid regulated MEX-6 mobility, we measured the average diffusivity of MEX-6 in the phospho-mutant strains at steady state. In the *mNG::mex-6(S403A)* mutant strain, the diffusivity dropped to a low, constant value of ~0.52 µm^2^/s (*SI Appendix*, Table S3), consistent with a lack of conversion of slow to fast MEX-6 by phosphorylation (Fig. 1*E*). This was confirmed in the *GFP::mex-6(S403A)* strain (*SI Appendix*, Fig. S1*D* and Table S3) and is consistent with previously published results for MEX-5 (21, 26).

At the anterior, dephosphorylated MEX-5 becomes slow (26, 34, 35, 38) by binding to RNAs via its CCCH ZF motifs (26). MEX-5 and MEX-6 share the tandem organization of their ZF domains (Fig. 1*A*). To address whether the mobility of MEX-6 was also affected by the formation of complexes with RNAs, we created a *mNG::mex-6(R277E; K321E)* mutant strain. Mutations of the conserved amino acids in *mex-5* (Fig. 1*A*) decrease the binding specificity of the protein to RNAs (33) and cause it to diffuse faster than in the control condition due to a lighter RNA burden (26). MEX-6(R277E; K321E) diffused almost two-fold faster than MEX-6 at both the anterior and posterior (Fig. 1*E* and *SI Appendix*, Table S3), similarly to the results published for the corresponding *mex-5* mutant (26). In the *mNG::mex-6(R277E; K321E)* strain, the mutant MEX-6 protein displayed a smoother gradient compared to the control (Fig. 1*F*), coherent with an imbalance between the slow and the fast components of the protein and the fact that we still detected a difference between anterior and posterior diffusivity. However, MEX-6(R277E; K321E) concentration was not as uniform as in the *S403A* mutant, as previously observed for the same mutations in the CCCH motifs of MEX-5 (26), suggesting that either MEX-6 can still bind some RNAs in vivo or that other yet unknown, additional mechanisms may contribute to the formation of the gradient.

These results show that MEX-6 forms a gradient through a differential switch of the protein diffusion state, similarly to MEX-5. However, in control conditions, MEX-6 diffusion is overall slower than that of MEX-5, both at the anterior and at the posterior.

### Monte Carlo Modeling of MEX-6 Differential-Diffusion Mechanism.

We wondered whether the slow diffusivity of MEX-6 was sufficient to establish the gradient by differential diffusion in the time window of the first cell division or whether this required yet unknown mechanisms. We tested this by using the computational framework that we developed in ref. 32 to simulate the molecular dynamics and the gradient formation of the protein MEX-5 and of the kinase PLK-1. We simulated how MEX-6 diffuses and forms a gradient, by modeling the random dynamics of its individual molecules starting from the experimental data collected by FRAP on the low average diffusivity of MEX-6. As the measurements performed in the *mNG::mex-6* and in the *GFP::mex-6* strains were not significantly different at the anterior (*SI Appendix*, Fig. S1*E*), for the calculations we considered an average anterior MEX-6 diffusivity of $D_{A,aver}$ = 0.55 µm^2^/s. At the posterior, the experimental diffusivity of mNG::MEX-6 was higher than that of GFP::MEX-6 (*SI Appendix*, Fig. S1*E*), we thus used an intermediate value of $D_{P,aver}$ = 0.91 µm^2^/s for MEX-6 posterior diffusivity, which is within the biological variability of the FRAP measurements. From these average values, we calculated the diffusion coefficients of the fast (phosphorylated by PAR-1) and slow (dephosphorylated and RNA-bound) components of MEX-6 (*SI Appendix*, Eqs. **S2** and **S3**). We obtained $D_{MEX-6,s}$ = 0.01 µm^2^/s and $D_{MEX-6,f}$ = 1.81 µm^2^/s (Table 1), finally converted through *SI Appendix*, Eq. **S4** to the velocities of the molecules to input in the simulations (Table 1).

**Table 1.** Parameters and assumptions used for the modeling of MEX-6 gradient and of MEX-5 gradient under different conditions for PAR-1 domain length

Symbol	Parameter	Value and source
$C_{A,MEX-6s}$ and $C_{A,MEX-5s}$	Relative concentration of slow MEX-6 at the anterior at steady state	70%, as measured in ref. 26 for MEX-5 and as used in ref. 32
$C_{A,MEX-6f}$ and $C_{A,MEX-5f}$	Relative concentration of fast MEX-6 at the anterior at steady state	30%, as measured in ref. 26 for MEX-5 and as used in ref. 32
$C_{P,MEX-6s}$ and $C_{P,MEX-5s}$	Relative concentration of slow MEX-6 at the posterior at steady state	50%, as measured in ref. 26 for MEX-5 and as used in ref. 32
$C_{P,MEX-6f}$ and $C_{P,MEX-5f}$	Relative concentration of fast MEX-6 at the posterior at steady state	50%, as measured in ref. 26 for MEX-5 and as used in ref. 32
$k_{PAR-1,low}$	PAR-1 phosphorylation rate, lower limit at steady state	0.001 s^−1^, as for MEX-5 in ref. 32
$k_{PAR-1,low}\times 1.5$	Lower limits of the PAR-1 kinase rate tested in Fig. 5 *D*–*F*	0.0015 s^−1^
$k_{PAR-1,low}\times 2$		0.002 s^−1^
$k_{PAR-1,low}\times 4$		0.004 s^−1^
$k_{PAR-1,upp}$		PAR-1 phosphorylation rate, upper limit at steady state
$k_{\mathrm{phosp}}$	Phosphatase rate	0.005 s^−1^ as for MEX-5 in ref. 32
$D_{MEX-6s}$ and $D_{MEX-6f}$	Diffusivity of slow and fast MEX-6	0.01 µm^2^ s^−1^, 1.81 µm2 s^−1^
$v_{MEX-6s}$ and $v_{MEX-6f}$	Velocity of slow and fast MEX-6	0.29 µm s^−1^, 3.80 µm s^−1^
$D_{MEX-5s}$ and $D_{MEX-5f}$	Diffusivity of slow and fast MEX-5	0.03 µm^2^ s^−1^, 3.00 µm2 s^−1^ as in ref. 32
$v_{MEX-5s}$ and $v_{MEX-5f}$	Velocity of slow and fast MEX-5	0.45 µm s^−1^, 4.90 µm s^−1^ as in ref. 32

**Fig. 2.**
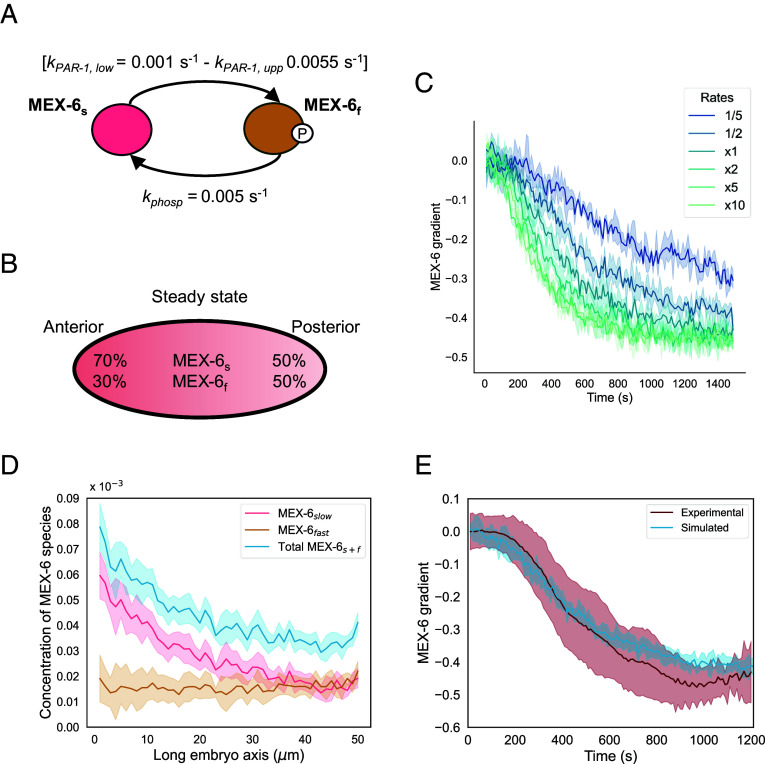
MEX-6 slow diffusivity allows the formation of a gradient through differential diffusion. (*A*) Summary of the interconversion rates between slow (dephosphorylated) MEX-6_s_ and fast (phosphorylated) MEX-6_f_. The phosphorylation rate by PAR-1 ($k_{PAR-1}$) has an anterior lower value of $k_{PAR-1,low}$ and a posterior value of $k_{PAR-1,upp}$. The PP2 phosphatase rate ($k_{\mathrm{phosp}}$) is constant throughout the embryo. The scheme reports the final values chosen for the simulation of MEX-6 gradient. (*B*) Scheme of a one-cell embryo showing the relative percentages of MEX-6_s_ and MEX-6_f_, at the anterior and posterior sides and at steady state. (*C*) Simulation of the dynamics of the MEX-6 gradient in scenarios where the values of three reaction rates shown in (*A*) are multiplied or divided by constant factors (as in the legend). The steady-state gradient is not impacted within the tested range, but only the dynamics of the process. (*D*) Concentrations of slow (pink), fast (orange), and total (blue) MEX-6 as a function of space along the embryo axis at steady state (t = 20 min). The concentrations were calculated in a central, 5-µm thick slice of a 3D ellipsoidal model of the one-cell embryo, by dividing the number of molecules of each MEX-6 species in each voxel by the total number of simulated particles. (*E*) Comparison of the kinetics of gradient formation of mNG::MEX-6 from the experimental results (bordeaux) and the simulations (blue). In (*C*–*E*), the solid lines represent the simulation results and are average values from at least five different simulation runs. For the experimental curve in (*E*), the curve is the average of 10 embryos. The shaded areas represent the SD.

We modeled the cycle of phosphorylation and dephosphorylation of the MEX-6 molecules by simulating the activity rates of the PAR-1 kinase ($k_{PAR-1})$ and of the phosphatase ($k_{\mathrm{phosp}})$ (Fig. 2*A*): the $k_{\mathrm{phosp}}$ was simulated as constant in space, while the PAR-1 kinase rate linearly increased from anterior to posterior (from $k_{PAR-1,low}$ to $k_{PAR-1,upp}$) (Fig. 2*A* and *SI Appendix*).

For the choice of the parameter to use for the simulations, we performed a parameter sensitivity analysis by fixing $k_{PAR-1,low}$ = 0.001 s^−1^ and modifying $k_{PAR-1,upp}$ and $k_{\mathrm{phosp}}$ (*SI Appendix*, Fig. S2*A*). Different combinations of the parameters altered the balance of slow/fast components and therefore the steady-state gradient of MEX-6 (*SI Appendix*, Fig. S2*A*), coherent with previous results for MEX-5 (24, 26). Our measurements in Fig. 1*B* revealed similar steady-state gradients for mNG::MEX-5 and mNG::MEX-6 in the control conditions. Therefore, we decided to use the same reaction rates that were shown to allow MEX-5 gradient formation (32) (Table 1, Fig. 2*A* and *SI Appendix*, Fig. S2*A*). Because of this, we assumed that the relative concentrations of the fast and slow MEX-6 species at steady state are the same to those measured for MEX-5 by fluorescence correlation spectroscopy in ref. 26 (Table 1 and Fig. 2*B*). As demonstrated in refs. 26 and 32, the factor determining the concentration steepness at steady state is the kinase-to-phosphatase rate ratio at the anterior and at the posterior. Results in Fig. 2*C* show that modifying the three reaction rates of a constant factor only perturbed the dynamics with which the steady state was reached (26, 32).

From the Monte Carlo model of MEX-6 dynamics, we obtained a good agreement between the simulated distribution of the MEX-6 protein compared to the experimental concentration observed by time-lapse microscopy in the one-cell embryo, both before gradient establishment and at steady state (*SI Appendix*, Fig. S2*B*). We then separately computed the distribution of fast and slow MEX-6 at steady state from anterior to posterior (Fig. 2*D*), showing that MEX-6 formed a concentration gradient due to the enrichment of slow MEX-6 at the anterior. The fast component of MEX-6 was instead homogenously distributed (Fig. 2*D*). These distributions were in agreement with those of MEX-5 in ref. 32. Quantification of the full kinetics of gradient formation from simulations showed a good agreement with the experimental data for early timepoints (Fig. 2*E*), but a less steep simulated gradient at steady state.

Based on these simulations, we conclude that despite MEX-6 has a lower diffusion coefficient than MEX-5, a differential-diffusion mechanism can still explain its gradient establishment in the time of the first division.

### Replacing the ZF Domains of the MEX-5 and MEX-6 Proteins Does Not Impact Their Diffusivity but Results in a Less Steep Gradient.

**Fig. 3.**
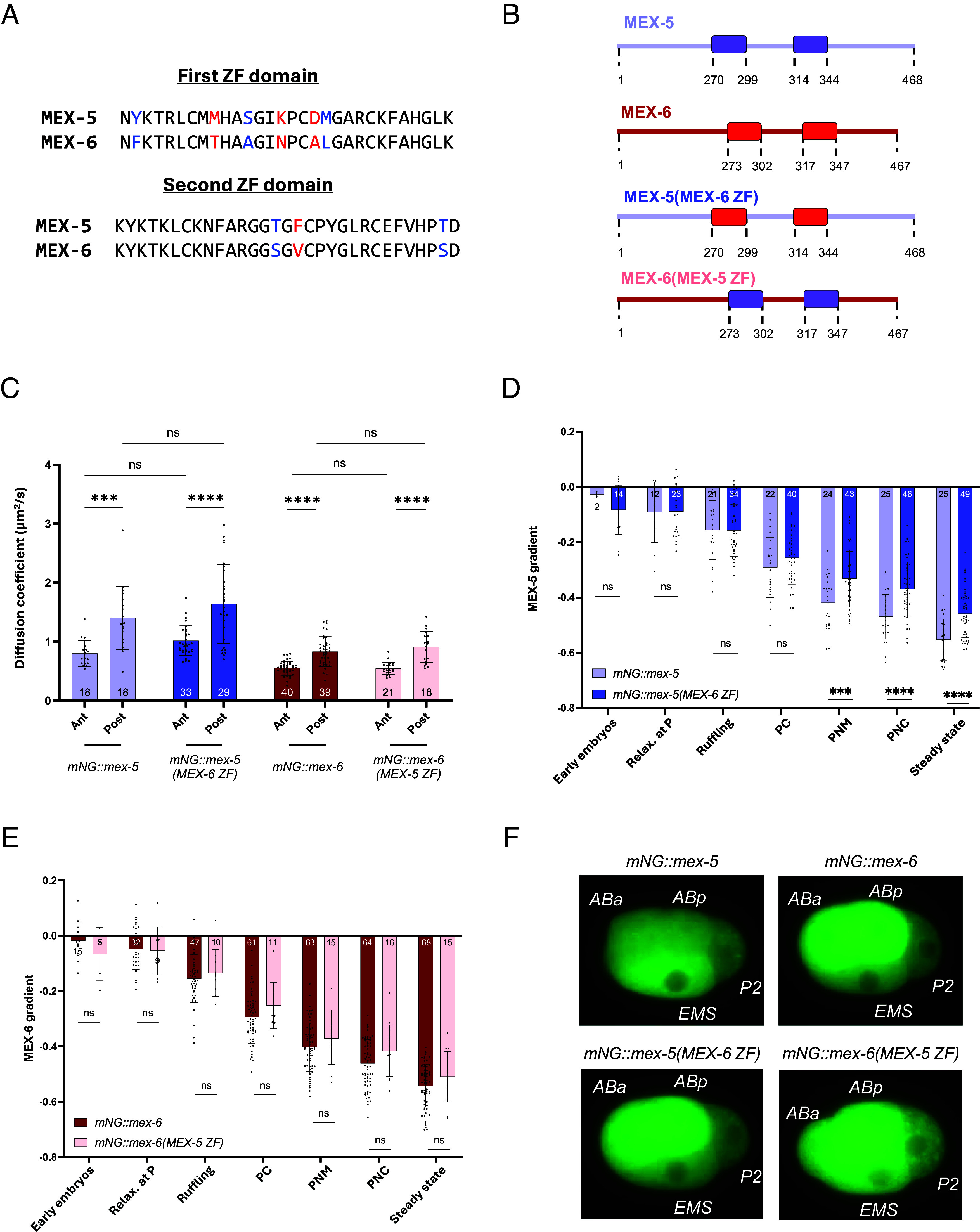
Replacing the ZF domains between MEX-5 and MEX-6 does not change their diffusivity but results in a less steep gradient. (*A*) Alignment of the first (*Top*) and second (*Bottom*) ZF domains of MEX-5 and MEX-6. It shows in black the identical amino acids, in blue the similar amino acids, and in red the amino acids that are not conserved. (*B*) Scheme of the design of the *mex-5(MEX-6 ZF)* and *mex-6(MEX-5 ZF)* mutant strains obtained by CRISPR. (*C*) Comparison of the diffusivity of mNG::MEX-5(MEX-6 ZF) with mNG::MEX-5 and of mNG::MEX-6(MEX-5 ZF) with mNG::MEX-6. The diffusivity was measured at anterior and posterior sides of steady-state embryos. The statistical analysis was performed using the two-way ANOVA test, with Tukey’s multiple comparison, independently for the two chimeric strains and their controls. (*D* and *E*) Comparison of the gradient of mNG::MEX-5(MEX-6 ZF) with that of mNG::MEX-5 (*D*) and of mNG::MEX-6(MEX-5 ZF) gradient with the mNG::MEX-6 gradient (*E*), as a function of time. The statistical analysis was performed, for each stage separately, using the two-tailed unpaired *t* test. (*F*) Images showing 4-cell stage embryos expressing mNG::MEX-5, mNG::MEX-6, mNG::MEX-5(MEX-6 ZF), and mNG::MEX-6(MEX-5 ZF) protein localization. The different daughter cells (ABa, ABp, P2, and EMS) are labeled in white. In (*C*–*E*), the bars represent the average values of the different measurements and the error bars the SD. The number N of analyzed embryos is reported for each condition.

As MEX-5 and MEX-6 are highly similar proteins (70% identity, 85% similarity, Fig. 1*A*), we asked what makes the diffusivity of the two proteins different. MEX-5 interacts with RNA complexes, which slows down its mobility (26). MEX-5 and MEX-6 ZF domains are similar to each other, with the first domain sharing 80% and the second domain 90% of identity (Fig. 3*A*), but Pagano et al. showed that the purified ZF domains of MEX-6 have a higher affinity for RNAs than those of MEX-5 (33). This raised the hypothesis that MEX-6 could bind to RNAs more efficiently than MEX-5, resulting in MEX-6 being on average slower than MEX-5 in the cytoplasm.

To address this hypothesis, we created a chimera strain where both the first and second ZF domains of MEX-5 were replaced by those of MEX-6 (Fig. 3*B*) in the *mNG::mex-5* background (*mNG::mex-5(MEX-6 ZF)* strain). This strain was viable and produced viable progeny (92.4% ± 5.3% vs. 94.3 ± 10.6% for the *mNG::mex-5* strain).

We asked whether the diffusivity of mNG::MEX-5(MEX-6 ZF) was reduced with respect to the parental *mNG::mex-5* strain. The average values of mNG::MEX-5(MEX-6 ZF) diffusivities at the anterior and at the posterior were not significantly different from the average values of mNG::MEX-5 and were rather higher than lower (Fig. 3*C* and *SI Appendix*, Table S3). These data suggest that the CCCH domains of MEX-6 are not the main determinant of its slow diffusivity.

We wondered whether the diffusivity could be decreased but not sufficiently to be detectable by FRAP due to the generally high diffusivity of MEX-5, in particular of its fast species (24, 26, 32). We therefore created the opposite chimera, *mNG::mex-6(MEX-5 ZF)* (Fig. 3*B*), to test whether the CCCH domains of MEX-5 could increase the diffusivity of MEX-6. This chimeric strain was also viable and produced viable progeny (97.97 ± 1.74% vs. 99.01 ± 1.56% for the *mNG::mex-6* strain). The difference in the average diffusivity of mNG::MEX-6(MEX-5 ZF) was not statistically significant from that of mNG::MEX-6 (Fig. 3*C* and *SI Appendix*, Table S3).

Despite the lack of major changes in diffusivity, the chimeric mNG::MEX-5(MEX-6 ZF) and mNG::MEX-6(MEX-5 ZF) had a trend to form less steep concentration gradients compared to mNG::MEX-5 and mNG::MEX-6 (Fig. 3 *D* and *E*). We also observed a difference in the behavior of mNG::MEX-5(MEX-6 ZF) and mNG::MEX-6(MEX-5 ZF) in later embryos. Wild-type MEX-5 is degraded in the somatic daughter cells ABa and ABp (21, 28, 34), and this degradation depends on its ZF domains (34) (Fig. 3*F* and Movie S2*A*). Despite having highly similar ZF motifs, MEX-6 was not degraded at this stage with its levels remaining high in ABa and ABp (Fig. 3*F* and Movie S2*B*). The chimeric MEX-5(MEX-6 ZF) behaved as MEX-6, with its levels remaining high in the 4-cell stage and in later embryos (Fig. 3*F* and Movie S2*C*). Vice versa, the chimeric MEX-6(MEX-5 ZF) showed a similar degradation pattern to MEX-5 (Fig. 3*F* and Movie S2*D*).

Our data suggest that in vivo, the reduced diffusivity of MEX-6 does not depend on an increased RNA affinity of its CCCH motifs, as MEX-5(MEX-6 ZF) is, if anything, slightly faster compared to MEX-5 at both sides of the embryo and MEX-6(MEX-5 ZF) has the same average diffusivity as MEX-6. However, the distribution of the chimeric proteins is altered in later embryos, and the morphology of their gradients at the one-cell stage is affected. This suggests that the reaction–diffusion mechanism, that is responsible for the gradient formation, may be backed up by processes that do not depend on the diffusivity and that are at the moment still uncharacterized.

### MEX-5, MEX-6, and PLK-1 Contribute to Each Other’s Gradient Formation.

**Fig. 4.**
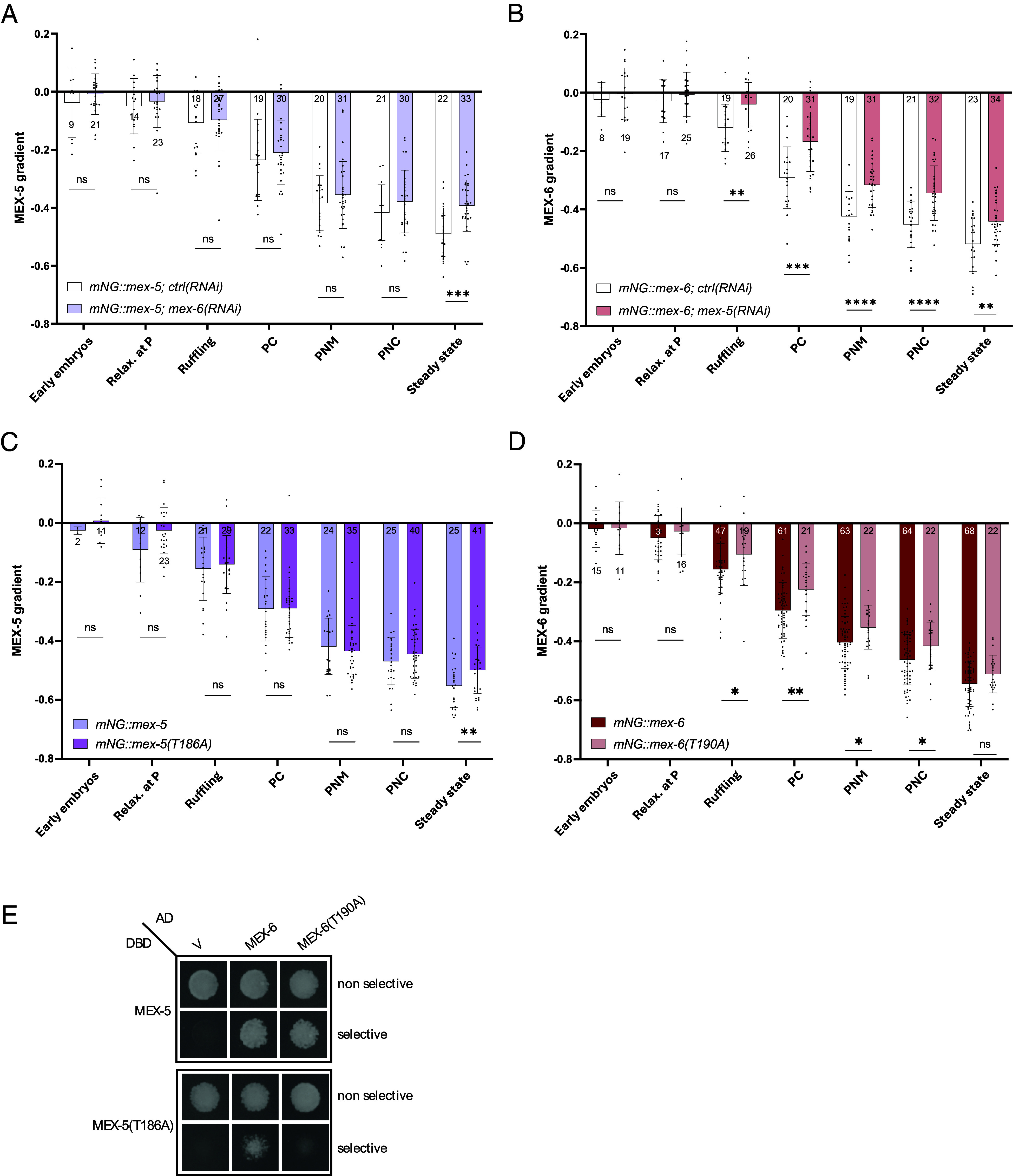
PLK-1 regulates MEX-5 and MEX-6 gradients and their interaction. Comparison of the dynamics of (*A*) MEX-5 gradient after treatment with *ctrl(RNAi)* and *mex-6(RNAi)* in the *mNG::mex-5* strain; (*B*) MEX-6 gradient after treatment with *ctrl(RNAi)* and *mex-5(RNAi)* in the *mNG::mex-6* strain; (*C*) MEX-5 gradient in the *mNG::mex-5(T186A)* mutant compared to the parental *mNG::mex-5* strain; (*D*) MEX-6 gradient in the *mNG::mex-6(T190A)* mutant compared to the parental *mNG::mex-6* strain. (*E*) Results of the yeast two-hybrid assay with the Gal4-DBD domain bound to the sequence of wild-type MEX-5 or MEX-5(T186A) and the Gal4-AD domain bound to wild-type MEX-6 or MEX-6(T190A), in both selective and nonselective medium. The empty vector containing the Gal4-AD domain only (V) was used as negative control. In (*A*–*D*), the statistical analysis was performed, for each stage separately, using the two-tailed unpaired *t* test. The bars represent the average values of the different measurements and the error bars the SD. The number N of analyzed embryos is reported for each condition.

Since MEX-5 and MEX-6 interact with each other (39), we hypothesized that the interaction between the two MEX proteins could be important to regulate their asymmetric distribution. Hence, we first investigated whether the depletion of one MEX protein influenced the gradient formation of the other. We performed *mex-6(RNAi)* (Fig. 4*A* and *SI Appendix*, Fig. S3*A*) using a dsRNA construct selectively targeting MEX-6 (28) and measured MEX-5 gradient. The gradient was less steep than the control starting from the pseudocleavage stage, but the impairment became significantly different only at steady state (Fig. 4*A*), in agreement with previous results (23). We then asked whether MEX-5 contributed to the formation of the gradient of MEX-6. We performed *mex-5(RNAi)* to specifically deplete the MEX-5 protein and found that also in this case, the gradient of MEX-6 was reduced compared to the control (Fig. 4*B* and *SI Appendix*, Fig. S3*B*), significantly starting from the ruffling stage until the steady state. These data suggest that directly or indirectly, MEX-5 and MEX-6 contribute to the formation of each other’s gradients.

MEX-5 and MEX-6 drive the formation of the gradient of the mitotic kinase PLK-1. Data from our lab and others showed that affecting the concentration gradient of one of the MEX proteins impacts the localization of PLK-1 (23, 25, 30–32, 36). We next asked whether PLK-1 could, at its turn, affect the MEX gradients. Mutation of one threonine in the PDS of MEX-5 (T186) and MEX-6 (T190) to alanine prevents the interaction with PLK-1 in yeast two-hybrid assays (30). We quantified the distribution of PLK-1 in the *mNG::mex-5(T186A)* and *mNG::mex-6(T190A)* mutants at the 2-cell stage as the ratio of PLK-1 signal in the anterior AB cell over the signal in the posterior P1 cell. We found that PLK-1 was less enriched at the anterior compared to the controls (*SI Appendix*, Fig. S4*A*), consistent with a lack of interaction of the MEX proteins with PLK-1 and the published literature (23, 30–32, 36). We then measured the kinetics of the MEX concentration gradients in these PDS mutants. For both MEX-5(T186A) and MEX-6(T190A), the steady-state gradients were smoother compared to those in the parental strains (Fig. 4 *C* and *D*). Looking at the temporal dynamics, in the *mNG::mex-5(T186A)* strain we did not detect any statistically significant difference in the concentration gradient before steady state (Fig. 4*C*), whereas in the *mNG::mex-6(T190A)* strain, the effect of the PDS mutations started to be detectable from the ruffling stage (Fig. 4*D*). We tested whether the smoothening of the gradient of MEX-5(T186A) and MEX-6(T190A) at steady state was correlated to a change in their diffusivity behavior. For both PDS mutants, we did not detect any significant difference compared to the control, with MEX-5(T186A) being only mildly faster than MEX-5 (*SI Appendix*, Fig. S4*B* and Table S3) and MEX-6(T190A) being mildly slower than MEX-6 (*SI Appendix*, Fig. S4*C* and Table S3).

Our results show that mutating the PDS of MEX-5 and MEX-6 and preventing the interaction with PLK-1 results in a weaker gradient of PLK-1 (as previously shown in refs. 23, 30–32, and 36) but also of the MEX proteins. This suggests that PLK-1 reinforces the anterior enrichment of both MEX proteins, which are in turn responsible for PLK-1 localization at the anterior in a feedback loop. For MEX-6, PLK-1 appears to regulate both the kinetics of gradient establishment (gradient defects appearing earlier) and the maintenance (less steep gradient). On the contrary, PLK-1 only affects the steady state of the MEX-5 gradient. The perturbations to the morphology of the MEX gradients are however not correlated to detectable changes of the mobility of MEX-5 and MEX-6 in the cytoplasm.

### PLK-1 Regulates MEX-5 and MEX-6 Gradients through Different Mechanisms.

As the lack of interaction between MEX proteins and PLK-1 affected the gradients of the formers without influencing their diffusivity, we then tested whether the mutagenized polo-docking sequences of MEX-5 and MEX-6 (T186A and T190A) affected the MEX-5/MEX-6 interaction by yeast two-hybrid assay. Results in Fig. 4*E* show that the mutation in the PDS of MEX-5 impaired the interaction with full-length MEX-6. The decrease in the gradient of MEX-5(T186A) (Fig. 4*C*) was similar to the decrease observed in the MEX-5 gradient when MEX-6 was depleted (Fig. 4*A*). This suggests that despite lacking the interaction with PLK-1 and partially with MEX-6, MEX-5(T186A) manages to form a gradient that becomes defective only at the steady state (Fig. 4*C*). Contrarily, MEX-6(T190A) was able to interact with full-length MEX-5 (Fig. 4*E*). We therefore suggest that the decrease in the gradient observed as a function of time for MEX-6(T190A) in Fig. 4*D* results from the missing interaction with PLK-1. When both the PDS of MEX-5 and MEX-6 were mutagenized, the interaction was completely prevented (Fig. 4*E*). Unfortunately, we could not study the behavior of the MEX proteins in vivo in the double PDS mutant as it is 100% embryonic lethal (28).

**Fig. 5.**
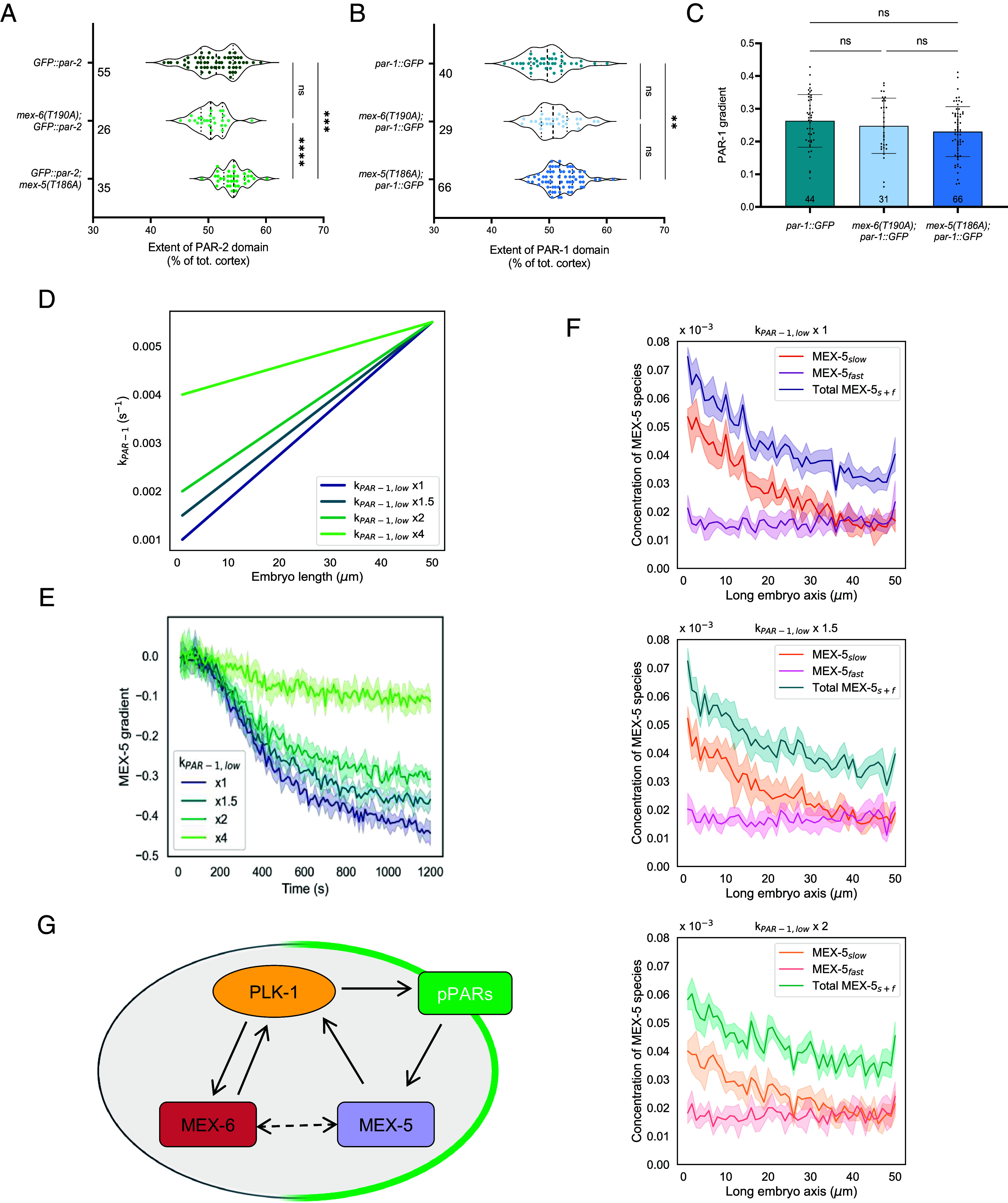
The *mex-5(T186A)* mutation influences cortical pPAR polarity. Quantification of (*A*) PAR-2 domain length in embryos of the *GFP::par-2, mex-6(T190A); GFP::par-2* and *GFP::par-2; mex-5(T186A)* strains; (*B*) PAR-1 domain length and (*C*) PAR-1 cytoplasmic gradient in embryos of the *par-1::GFP, mex-6(T190A); par-1::GFP* and *mex-5(T186A); par-1::GFP* strains. The domain length was calculated as the percentage of the total cortical length that is occupied by the domain. The quantifications were performed at stages after NEBD. (*D*) Plot of the different distributions of $k_{PAR-1}$ that were tested to reproduce a longer PAR-1 domain toward the anterior. The upper value $k_{PAR-1.upp}$ was kept fixed to 0.0055 s^−1^, while the value of $k_{PAR-1.low}$ was changed as reported in the legend. (*E*) Comparison of the simulated MEX-5 gradients as a function of time, for the different conditions of $k_{PAR-1,low}$ shown in (*D*). (*F*) Steady-state concentrations of slow (shades of orange), fast (shades of violet), and total [shades of viridis as in panel (*E*)] MEX-5 along the embryo axis, simulated for the values of $k_{PAR-1.low}$ specified above the graphs. The concentrations were calculated in a central 5-µm thick slice of a 3D ellipsoidal model of the one-cell embryo by dividing the number of molecules of each MEX-5 species in each voxel over the total number of simulated particles. (*G*) Model of the feedback circuits involving MEX-5, MEX-6, and PLK-1. PLK-1 regulates the gradient of MEX-6 directly, while it modulates MEX-5 gradient formation through a feedback loop involving cortical polarity. The interaction between MEX-5 and MEX-6 (dashed line) was shown in ref. 39 and in this work, but the reciprocal effect on their gradients could be indirectly regulated by PLK-1. In (*A* and *B*), the violin plots report the median (dashed line) and the quartiles (dotted lines) of the distributions. The individual dots are also displayed and the number N of analyzed embryos is reported for each condition. The statistical analysis was performed using the Kruskal–Wallis test, with Dunn’s multiple comparison. In (*C*), the bars represent the average values of the different measurements and the error bars the SD. The number N of analyzed embryos is reported for each condition. The statistical analysis was performed using the one-way ANOVA test, with Tukey’s multiple comparison. In (*E* and *F*), the solid curves represent the simulation results and are the average values from five different simulation runs. The shaded area represents the SD.

We next aimed to unravel whether PLK-1 influenced the gradient of MEX-5 through different mechanisms. For instance, perturbation of PLK-1 activity or expression was shown to perturb establishment and maintenance of the PAR polarity (40–45), and depletion of MEX-5/MEX-6 by RNAi also affected cortical polarity (3, 11, 28). We asked whether the PDS mutations, which created a smoother asymmetry of PLK-1 (*SI Appendix*, Fig. S4*A*), were sufficient to undermine the establishment of cortical polarity in the *mNG::mex-5(T186A)* and in the *mNG::mex-6(T190A)* strains. To address this question, we crossed the *mex-5(T186A)* and the *mex-6(T190A)* strains with a strain expressing a GFP-fusion of one of the posterior PAR proteins, PAR-2, and quantified the PAR-2 domain length after the pronuclear centration stage. We found that the PAR-2 domain in the *mex-6(T190A); GFP::par-2* strain was not different compared to that in the *GFP::par-2* strain (Fig. 5*A*). Instead we detected a longer PAR-2 domain in the *GFP::par-2*; *mex-5(T186A)* strain than in the control (Fig. 5*A*).

As PAR-2 drives the recruitment of the PAR-1 kinase at the posterior cortex (4, 5), a perturbation in PAR-2 distribution could entail a change of the PAR-1 phosphorylation rate regulating the switch between MEX-5 diffusion states (21, 22, 26). In this scenario, the morphology of MEX-5 cytoplasmic gradient could also be affected. Thus, we quantified the distribution of PAR-1 at the cortex crossing the *mex-5* and *mex-6* PDS mutant strains with an endogenously labeled PAR-1 strain (*par-1::GFP::par-1 exon11a*, hereafter called *par-1::GFP*). The difference in PAR-1 cortical localization in between the *par-1::GFP* and the *mex-6(T190A); par-1::GFP* strains was not statistically significant, despite a slight increase in the domain extension in the latter (Fig. 5*B*). The PAR-1 domain was instead longer in the *mex-5(T186A); par-1::GFP* strain than in *par-1::GFP* embryos (Fig. 5*B*), consistent with the increase in the PAR-2 domain size. In addition to its cortical localization, PAR-1 also displays an important cytoplasmic pool (37), which is essential to regulate MEX-5 gradient (26). Therefore, we quantified the PAR-1 concentration gradient in the cytoplasm. We could not detect a significant difference in the gradient between the PDS mutants and the control. However, the gradient displayed lower absolute values in both PDS mutants, but more in the *mex-5(T186A); par-1::GFP* strain (Fig. 5*C*).

To analyze the effects of a longer PAR-1 domain and of a consequent smoother cytoplasmic (activity) gradient on the steady-state gradient of MEX-5, we resorted to the Monte Carlo simulations. All the parameters related to MEX-5 reaction–diffusion mechanism refer to our previous work (32) (Table 1). To achieve a smoother PAR-1 gradient, we ran the simulations by increasing the value of $k_{PAR-1,low}$ of 1.5-, 2-, and 4-fold (Fig. 5*D* and Table 1). We found that the MEX-5 gradient decreased proportionally with the increase in $k_{PAR-1,low}$ (Fig. 5*E*), till an almost uniform protein distribution was reached for the value $k_{PAR-1,low}\times 4$, as suggested by gradient values close to zero (Fig. 5*E*). We obtained from the computations the distributions of the slow and fast species of MEX-5 in the control condition ($k_{PAR-1,low}\times 1$) and in the two scenarios where $k_{PAR-1,low}$ was multiplied by a factor of 1.5 and 2, and showed that the main effect of this parameter change was a decrease in the percentage of the dephosphorylated (slow) component of MEX-5 at the anterior (Fig. 5*F*). This resulted in a less steep concentration gradient for total MEX-5 (Fig. 5*F*), consistent with what measured in the *mNG::mex-5(T186A)* strain (Fig. 4*C*).

Our results show that mutating the PDS of MEX-5 (but not the one of MEX-6) results in altered cortical polarity, as detected from the localization of two posterior PAR proteins, PAR-1 and PAR-2. As the perturbation of the PAR-1 kinase distribution could reduce the asymmetry of the MEX-5 cytoplasmic gradient, we modeled a smoother PAR-1 gradient and looked at the one of MEX-5 as readout. The simulations support the hypothesis that deviations from normal cortical polarity trigger changes in downstream cytoplasmic gradients, as previously shown (46).

## Discussion

Polarity is a complex and dynamic process that is supported and maintained by the interplay of different proteins and mechanisms, ensuring its robustness. While the PAR proteins initially redistribute on the cortex to then drive the segregation of cytoplasmic factors (11, 22–24, 26–28), it has been showed that the maintenance of cortical polarity is controlled by proteins like MEX-5, MEX-6, and PLK-1 (11, 14, 28, 36, 40, 43, 47). However, how these proteins cross talk with each other and with the cortex is still not fully elucidated.

MEX-5 and MEX-6 are paralogs, sharing 70% identity and 85% similarity (Fig. 1*A*), with MEX-5 having an essential role in the development of the *C. elegans* embryo (11). In this study, we compared the biophysical properties of MEX-5 and MEX-6 and the mechanisms through which they establish a gradient. While we show that the MEX-6 gradient is governed by a reaction–diffusion mechanism similar to that of MEX-5 (Fig. 1 and *SI Appendix*, Fig. S1 for the GFP-tagged strains), MEX-5 and MEX-6 substantially differ in terms of average diffusivity in the cytoplasm, with the latter being slower than MEX-5 (half of MEX-5 diffusivity, Fig. 1*C* and *SI Appendix*, Fig. S1*B*). Our first question was whether such a slow diffusivity can ensure the formation of gradients by differential diffusion in the temporal constraint of a cell division, or whether redundant pathways need to be in place. We addressed this computationally, within the assumption that the relative steady-state concentrations of slow and fast components of MEX-6 were equal to those of MEX-5, experimentally measured in ref. 32. This assumption is justified by the fact that in a two-component model with fast and slow molecules, their relative concentrations at the anterior and posterior depend on the interconversion (here phosphatase and kinase) rates, and on their ratio at anterior and posterior (24, 26). Any change in the ratio would consequently affect the steady-state gradient. The mNG::MEX-6 concentration gradient resembles, in the control conditions, the one of mNG::MEX-5 (Fig. 1*B*), suggesting that the reaction rates and the steady-state relative abundances of slow and fast species of MEX-5 and MEX-6 are similar. We here show that forming a gradient in the time of the first division is theoretically possible without the involvement of any additional mechanism, despite the low molecular diffusivity (Fig. 2 and *SI Appendix*, Fig. S2). Under the assumptions regarding the used simulation parameters, the steady-state gradient of MEX-6 is found to be less steep than the experimental one (Fig. 2*E*). This suggests that the selected range of phosphorylation and dephosphorylation rates might only approximate the in vivo reaction kinetics.

Why do the two MEX proteins differ in diffusivity, despite their high homology? MEX-6 could be slower due to an enhanced affinity to RNAs of its ZF motifs, as suggested in ref. 33. The higher affinity could manifest in 1) single molecules of MEX-6 binding more RNA molecules than MEX-5, and/or 2) more MEX-6 molecules being more prone to bind (the same amount of) RNAs, increasing the fraction of MEX-6 in the slow state compared to that in the fast state. Both scenarios would lead to a general deceleration of MEX-6 compared to MEX-5 in the cytoplasm. According to this hypothesis, we expected that a chimeric form of MEX-5 presenting the ZF domains of MEX-6 (Fig. 3*B*) would show a decrease in the absolute value of the diffusivity. This is excluded by our FRAP measurements (Fig. 3*C*), although we could not exclude potential competition between the chimeric MEX-5(MEX-6 ZF) and MEX-6 for the same pool of RNAs, thus decreasing the available substrate per protein. Another possibility is that the introduction of the ZF domains into a different recipient alters their affinity to RNAs. Similarly, a MEX-6(MEX-5 ZF) chimera (Fig. 3*B*) did not show any increase in the diffusivity (Fig. 3*C*). Why MEX-6 diffuses slower remains so far unclear.

The gradients of the two chimeric MEX-5 and MEX-6 proteins displayed a general trend to be smoother with respect to their wild-type version (Fig. 3 *D* and *E*). This could be due to an increase in pool of fast molecules (even if against our hypothesis), as could be suggested by the mild increase in diffusivity of the chimeric proteins (*SI Appendix*, Table S3). Alternatively, it could suggest that the theoretical model of differential diffusion that allows the formation of the intracellular gradients of MEX-5 and MEX-6 could be backed-up by processes that appear to be diffusivity-independent.

To explore whether the gradient establishment could be supported by molecular cross talk, we shifted our attention to the physical and genetic interaction between MEX-5, MEX-6, and PLK-1. Preventing the binding to PLK-1 perturbs the MEX-5 and MEX-6 gradients, as shown in the PDS mutants (Fig. 4 *C* and *D*). Interestingly, the impairment in the gradients is not related to a detectable change in the diffusivity of the MEX PDS mutants (*SI Appendix*, Fig. S4 *B* and *C*), reinforcing the likelihood that diffusion may be not the only parameter of relevance for the mechanism of intracellular patterning, but that other still-unknown biophysical pathways could underlie cell polarity.

If we compare conditions where MEX-5 and PLK-1 cannot interact (either when MEX-5 is depleted—Fig. 4*B*—or mutated at the PDS—Fig. 4*C*), we observed that the MEX-6 gradient is impaired at earlier stages (Fig. 4*B*), whereas MEX-5 gradient itself is reduced only at the steady state (Fig. 4*C*). When MEX-6 and PLK-1 cannot interact (Fig. 4 *A* and *D*), the MEX-6 gradient is significantly reduced over time (Fig. 4*D*), while there is only a mild reduction of the MEX-5 gradient at steady state (Fig. 4*A*). These findings suggest a divergence between how PLK-1 regulates the gradients of MEX-5 and MEX-6 (Fig. 5*G*). Considering the timing at which the differences in the gradient appear in the PDS mutant of MEX-6, we postulate a stronger, direct regulation by PLK-1 on MEX-6 (Fig. 5*G*). Conversely, the late, mild divergence of the gradient of MEX-5 PDS mutant compared to control suggests that MEX-5 gradient is regulated by PLK-1 in an indirect way. We here hypothesize the mediation of a feedback loop involving cortical polarity (Fig. 5*G*). This is supported by the longer domains of the posterior PAR proteins PAR-1 and PAR-2 in the *mNG::mex-5(T186A)* strain at steady state (Fig. 5 *A*–*C*), which corresponds to the stage when the gradient of MEX-5(T186A) appears different (Fig. 4*C*). Conversely, the PAR protein localization in the *mNG::mex-6(T190A)* strain is not significantly different from the control at steady state (Fig. 5 *A*–*C*).

In conclusion, our results pave the way to a model where PLK-1 feedbacks to MEX-5 and MEX-6 through two distinct circuitries, which are essential to faithfully maintain polarity in the cytoplasm and at the cortex. Moreover, our model suggests that the communication between MEX-5 and cortical polarity is mediated by the PLK-1 kinase (Fig. 5*G*), as in the *mex-5(T186A)* mutant the pPARs are altered (Fig. 5 *A*–*C*). This work lays the groundwork to the understanding of how protein cross talk accounts for the correct establishment of patterning in the cytoplasm.

## Materials and Methods

One-cell *C. elegans* embryos where MEX-5 and MEX-6 (and mutants) were endogenously tagged with fluorophores were imaged by epifluorescence time-lapse microscopy to study the protein dynamics and gradient formation under control and RNAi conditions. The proteins’ average diffusivity was obtained by means of FRAP microscopy. Interaction between proteins was tested by yeast two-hybrid assay. The modeling of MEX-6 and MEX-5 gradients relied on the Monte Carlo simulations developed in ref. 32, extended to test the hypotheses addressed in this work. Details on the experimental and modeling procedures, on the strains, and on the reagents used in this study are provided in *SI Appendix*. Information on the analysis algorithms and pipelines and on the statistical analysis is also provided in *SI Appendix*.

## Supplementary Material

Appendix 01 (PDF)

Movie S1. **MEX-6 and MEX-5 gradient establishment in the one-cell *C. elegans* embryos**. Time-lapse movies of the first division of one-cell embryos of the *mNG::mex-6* (A) and of the *mNG::mex-5* (B) strains.

Movie S2. **Degradation patterns of the MEX-5 and MEX-6 proteins are different and depend on the proteins’ ZF domains**. Time-lapse movies of the cell divisions from the 4-cell stage embryos of the *mNG::mex-5* (A) and of the *mNG::mex-6* (B) strains reveal different degradation rates for the two proteins, with MEX-5 degraded in somatic cells at late 4-cell stage. In the chimera where MEX-5 ZF domains were replaced with those of MEX-6 (*mNG::mex-5(MEX-6 ZF)*) (C), MEX-5(MEX-6 ZF) levels remained high in the soma cells, with an homogeneous distribution in the embryo similar to wild-type MEX-6. Vice versa, in the chimera *mNG::mex-6(MEX-5 ZF)* (D), MEX-6(MEX-5 ZF) is degraded in the somatic cells similarly to wild-type MEX-5.

## Data Availability

The code for MEX-6 and MEX-5 gradient simulations is available at https://github.com/sofiabarbieri/MCeleM6 (48). The ImageJ and Python scripts for the analysis of time-lapse movies for the quantification of the gradient are available at https://github.com/sofiabarbieri/GradientAnalysis (49). The QuPath and MATLAB scripts for the analysis of PAR-2 cortical domains are available at https://github.com/sofiabarbieri/CorticalDomain (50). The Jupiter notebooks to analyze the data from FRAP microscopy are available at https://github.com/sofiabarbieri/FRAP_AnalysisTool (51). All the raw data are available in the Yareta depository: https://doi.org/10.26037/yareta:kceyprc4mvdvdmsdlk2dh547ku (52).
