# Supplementary material for: Internal feedback circuits among MEX-5, MEX-6, and PLK-1 maintain faithful patterning in the Caenorhabditis elegans embryo
Source: Proc Natl Acad Sci U S A. 2024 Dec 17;121(52):e2407517121. doi: 10.1073/pnas.2407517121 (PMC11670235; doi:10.1073/pnas.2407517121)
Supplement: Supplementary file 1 — Appendix 01 (PDF) [file pnas.2407517121.sapp.pdf]

Supporting Information for

Internal feedback circuits among MEX-5, MEX-6 and PLK-1 maintain faithful patterning in the *Caenorhabditis elegans* embryo

Alexandre Pierre Vaudano[1], Françoise Schwager[1], Monica Gotta[1,2,*], Sofia Barbieri[1,2,*]

[1] Department of Cell Physiology and Metabolism, Faculty of Medicine, University of Geneva, rue Michel-Servet 1, 1211 Geneva (CH)

[2] Co-last authors

* Co-corresponding authors: Dr. Sofia Barbieri, Prof. Monica Gotta

Emails: sofia.barbieri@unige.ch; monica.gotta@unige.ch

This PDF file includes:

Supporting text

Figures S1 to S4

Tables S1 to S6

Legends for Movies S1 to S2

SI References

Other supporting materials for this manuscript include the following:

Movies S1 to S2

Supporting Information Text

Material and Methods

Strain and Maintenance

The strains used in this work are listed in Table S1 and were maintained at 20°C according to standard protocols (1). The oligos used for the CRISPR strains originated in this work are listed in Table S2.

The *GFP::par-2; mex-5(T186A)* (ZU370) and *mex-6(T190A); GFP::par-2* (ZU371) strains were obtained by crossing *GFP::par-2* (KK1273) males with respectively *mex-5(T186A)* (JH3405) *and mex-6(T190A)* (PHX4753) hermaphrodites.

The *mex-5(T186A); par-1::GFP* (ZU376) and *mex-6(T190A); par-1::GFP* (ZU375) strains were obtained by crossing *par-1::GFP* (KK1262) males with respectively *mex-5(T186A)* (JH3405) *and mex-6(T190A)* (PHX4753) hermaphrodites.

Males were obtained by heat-shocking L4 worms and young adults at 30°C for 5h.

Some ZU370 and ZU376 worms displayed sterility, but the others produced viable progeny. All other strains used were viable and produced viable progeny.

Embryonic viability

Ten worms at the L4 stage were singled in small OP50 plates and left to lay embryos at 20°C for 24 hours, after which the hermaphrodites were removed from the plates. After 24 hours, the number of viable larvae and of unhatched embryos was counted, and the embryonic viability was determined as the number of larvae over the total brood size (larvae + unhatched embryos).

RNA interference

Single colonies of HT115 bacteria containing either the control *L4440* empty vector (“*ctrl(RNAi)*” in the Figures) or the plasmids for specific *mex-5(RNAi) (2)* or *mex-6(RNAi)* (2) were inoculated in LB medium supplemented with 100 μg/ml carbencillin and 10μg/ml tetracycline. Cultures were grown at 37°C under agitation to mid-log phase, then induced in 1 mM Isopropil-β-D-1-tiogalattopiranoside (IPTG) for 1 h at 37°C. Bacteria were seeded on 3 mM IPTG plates. Worms were fed from the L1 stage at 20°C, for 72 hours.

Live imaging conditions

Worms were dissected in Egg Buffer (118 mM NaCl + 48 mM KCl + 2 mM $CaCl_2$ + 2 mM $MgCl_2$ + 25 mM HEPES, pH 7.6) and the embryos were mounted on 3% noble agar pads before imaging.

For time-lapse recordings, a Nikon ECLIPSE Ni-U microscope, equipped with a Nikon DS-U3 Digital Camera, was used. Embryos where MEX-5 and MEX-6 (and their mutant forms) were tagged with GFP and mNG were exposed for 200-400 ms, while embryos of the *GFP::par-2* and of the *par-1::GFP* strains (and related crosses) were exposed for 600 ms and 800 ms respectively, with a fixed gain of 10%. For the DIC channel, an exposure time of 30 ms was used (35% intensity). Embryos were imaged in a room maintained at 20°C. For the study of the dynamics of gradient formation, one-cell embryos at different stages were selected and imaged till cytokinesis. For Fig. 2E, only early embryos before cortical relaxation and pronuclei appearance were analyzed, for a total duration of ~20 min with a time-frame every 10 s. For the assessment of MEX-5 and MEX-6 degradation patterns, the divisions of the embryo from four-cell stage to multicellular stage were acquired for ~40 minutes, with frames taken every minute (Fig. 3F, Supplementary Movies S2). For the analysis of PAR-1 cytoplasmic gradient (Fig. 5C), one-cell embryos were analyzed at stages from NEBD.

FRAP movies were acquired on a confocal Nikon A1r Spectral microscope (Ti Eclipse inverted stand), used in Galvano mode and equipped with a 60x 1.4 CFI Plan Apo Lambda (WD: 0.13mm) oil-immersion objective. A single circular ROI of 2.5-µm radius was drawn either in the anterior or in the posterior of the embryo, close to the cortex. Pre- and post-FRAP imaging was carried out using a 488-nm laser, at a 9-15% laser power (according to the strain intensity). A 256 x 256 pixel resolution and a scan speed of 0.25 s/frame were used. An electronic 5-fold magnification was applied. Five frames were acquired before bleaching to evaluate the intensity baseline. The ROI was bleached using simultaneously a 405 and a 488 nm laser (100% laser power, scan speed = 0.25 s/frame) and then recorded for ~1 min to evaluate the signal recovery. Embryos at stages later than pronuclear centration were selected and imaged in a room maintained at 22°C.

Gradient analysis

The quantification of the gradient as a function of time from epifluorescent time-lapse movies was performed as in (3) using a ImageJ macro (4), available on GitHub (see section Data, Materials, and Software Availability). The MEX-5, MEX-6 and PAR-1 intensity in one-cell embryos was extracted from a line ROI crossing the cytoplasm from the highest protein concentration to the lowest (anterior to posterior for MEX-5 and MEX-6, posterior to anterior for PAR-1), avoiding the pronuclei and the cortex. The intensity profile was obtained for each frame of the time-lapse movie, and further processed using a custom Python script (see section Data, Materials, and Software Availability) to normalize the intensity to the maximum value. The x-axis was also normalized to 1 based on the length of the line ROI. The concentration profiles were fitted with a linear function, from which the linear coefficient was extracted as a measurement of the gradient. For PAR-1, the inverse values of the gradients are plotted to maintain the convention anterior-to-posterior.

Embryos of the same strain/condition from different experiments were pooled together and averaged, and the results were presented as mean ± SD.

Measurement of the diffusivity

The diffusion coefficient (D_c) was calculated using the following equation S1 (5):

$$D_\mathrm{c} = {(r_n^2 + r_{eff}^2)} \Big/ {8 \cdot \tau_{1/2}} \qquad \text{Eq. S1}$$

with $\tau_{1/2}$ the half-time of recovery, r_n the nominal radius of the bleached area (2.5 μm), and r_{eff} the effective bleached radius (5). The latter was defined as the radius at 86% of the height of a Gaussian fitting the signal over the bleached ROI in the first post-FRAP timeframe (3, 5). For the quantification of $\tau_{1/2}$, after subtraction of the background signal, the curves were normalized to zero by subtracting the intensity in the first post-FRAP frame, and to the average baseline signal pre-FRAP. The curves were corrected for photo-bleaching due to the imaging and finally fitted using a 1-phase association, non-linear regression function to obtain $\tau_{1/2}$ (3). Embryos of the same strain/condition from different experiments were pooled together and averaged, and the results were presented as mean ± SD.

Table S3 reports the average value and the associated standard deviation (SD) of the diffusivities measured in this work.

Immunofluorescence and image acquisition

For the quantification of PLK-1 asymmetry (Fig. S4A), adult hermaphrodites were dissected in 10 µl of M9 buffer (86 mM NaCl, 42 mM Na2HPO4, 22 mM KH2PO4, and 1 mM MgSO4) on an epoxy slide square (Thermo Fisher Scientific), previously coated with 0.1% poly-*L*-lysine. The embryos were mechanically permeabilized by being squeezed with a 22 × 40 mm coverslip, then transferred onto dry ice for 10 min before flicking off the coverslip. The samples were fixed for 20 min in methanol at room temperature and then blocked for 20 min in PBS + 0.2% Tween20 (PBST) with 1% BSA. Samples were incubated with the primary α-PLK-1 antibody (6) (rabbit, 1:500 in PBST) overnight at 4°C and washed twice for 10 min in PBST. The slides were incubated for 45 min at 37°C with 4 µg/ml of Alexa Fluor 568 secondary antibody and 1 µg/ml DAPI in PBST. Samples were washed twice for 10 min in PBST and mounted with Mowiol (30% (w/v) glycerol, 3.87mM Mowiol (Calbiochem 475904), 0.2 M Tris-HCl pH 8.5, 0.1% DABCO).

The intensity of PLK-1 from two square ROIs in the cytoplasm of the anterior AB cell and the posterior P1 cell was averaged, and the signal asymmetry calculated as the ratio of the signal in AB to the signal in P1.

Quantification of PAR-2 and PAR-1 domain length

The cortical domain of PAR-2 (Fig. 5A) was quantified using *ad-hoc* analysis scripts for QuPath 0.5.1 (7). The script used an Artificial Neural Network Multilayer-Perceptron Pixel Classifier (OpenCV) in QuPath. The Pixel Classifier was trained on around 30 annotated images of GFP::PAR-2 from embryos of different strains/conditions, using 6 features for the training ("Gaussian", "Laplacian", "Weigthed_Std_Dev", "Gradient_Magnitude", "Structure_Tensor_Coherence", "Hessian_Eigenvalue_Min").

Detected regions with an area smaller than 5 μm^2 were discarded (to avoid detection of, *e.g.,* the nuclear body). For the detection of the whole embryonic cortex, we used Cellpose2.0 (8), with a model custom-trained on the DIC images of 35 embryos. Following the detection of both the embryo cortex and of the PAR-2 domain, a script in MATLAB (MATLAB R2024a, The MathWorks), provided the percentage of the total cortex occupied by the PAR-2 domain. The scripts are available on GitHub (see section Data, Materials, and Software Availability).

The cortical domain of PAR-1 (Fig. 5B) was not recognizable by the QuPath algorithm due to the high cytoplasmic pool of PAR-1, which prevented the domain from being

automatically segregated from the cytoplasm. The domain was therefore detected manually.

Cloning of MEX-5 and MEX-6 sequences and site directed mutagenesis

The MEX-5 and MEX-6 sequences were cloned into the pDONR201 vector and the T186A mutation in MEX-5 and theT190A mutation in MEX-6 were inserted by site-directed mutagenesis. The oligos used to clone the sequences and to perform the side-directed mutagenesis are listed in Table S4.

MEX-6 and MEX-6(T190A) were then transferred to the pDEST22 vector (Gal4-AD domain) and MEX-5 and MEX-5(T186A) to the pDEST32 vector (Gal4-DBD domain) using the Gateway technology, as the interaction between MEX-5 and MEX-6 was detected in this direction (9). Plasmids used in this study are reported in Table S5.

Yeast two-hybrid

The two-hybrid interaction was assessed using the Gal4-based system (Gateway, Invitrogen), using the MAV203 yeast strain. MAV203 yeast were transformed with 1 µg of Gal4-DBD and Gal4-AD DNA combinations and the colonies let to grow for 3 days at 30°C. Transformants were selected on synthetic-defined medium lacking leucine and tryptophan (*"non selective"* in the Figures). Two colonies of each transformation condition were dissolved into 100 µl dH_2O and serial dilutions prepared to be spotted onto plates lacking leucine, tryptophan, and histidine and containing 25 mM 3AT (3-amino-1,2,3-triazole; Sigma-Aldrich) (*"selective"* in the Figures) and onto *"non selective"* plates as control. The plates were kept at 30°C for 3 days and then imaged using the Fusion FX6 EDGE Imaging System (Vilber) equipped with an Evo-6 Scientific Grade CCD camera, available at the Faculty of Medicine.

Monte Carlo simulations

Simulations of MEX-6 gradient establishment were performed using the framework developed in (3). The 1-cell *C. elegans* embryo was simulated as a 3D ellipsoid (50 × 30 × 30 µm³ along x, y, and z) and the molecules as point-like structures. At the beginning of the simulations, MEX-6 was uniformly distributed, with relative concentrations of 70% MEX-6_s and 30% MEX-6_f, as experimentally measured for MEX-5 (10). 10^6 molecules were generated for each simulation and made to diffuse in the volume by Brownian motion, with velocities matching the experimental diffusion

coefficients (see next paragraph). The change of MEX-6 diffusivity depended on the PAR-1 kinase and the PP2A phosphatase rates. The PP2A phosphatase activity (k_{phosp}) was modeled as uniformly distributed in the cytoplasm (Fig. 2A). PAR-1 activity was simulated as constant and low ($k_{\mathrm{PAR-1,low}}$) at the beginning of the simulations, to then gradually increase in the posterior over the first 300 seconds. This created an anterior-low ($k_{\mathrm{PAR-1,low}}$) to posterior-high ($k_{\mathrm{PAR-1,high}}$) activity gradient (Fig. 2A). The state switch and the Brownian diffusion were iteratively simulated for each particle every second. Since in epifluorescence microscopy the light was detected from a wide portion of the sample, the particle concentrations were calculated by projecting onto a 2D plane the molecules present in a 5-µm-thick slice centered around the middle plane of the volume (3). We extracted the number of particles along a line crossing the cytoplasm from anterior to posterior, every 10 s, as done experimentally, and the MEX-6 concentration profiles were fitted with a linear function, to compute the gradient (3).

The simulations in Fig. 5D-F were obtained by using the parameters related to MEX-5 reaction-diffusion model and diffusivity reported in (3) (see section Data, Materials, and Software Availability). To analyse the effect of a flatter kinase rate on the MEX-5 gradient, the lower limit of the PAR-1 rate $k_{PAR-1,low}$ was increased of 1.5-, 2- and 4-folds.

The input parameters for the MEX-6 and MEX-5 simulations are listed in Table 1 in the Main Text.

Calculation of the input diffusivity parameters for the simulations

To calculate the diffusivity of the fast and slow components of MEX-6 ($D_{\mathrm{MEX-6,f}}$ and $D_{\mathrm{MEX-6,s}}$), we used the following equations (3):

$$D_{\mathrm{A,aver}} = C_{\mathrm{A,MEX-6f}} \cdot D_{\mathrm{MEX-6f}} + C_{\mathrm{A,MEX-6s}} \cdot D_{\mathrm{MEX-6s}} \qquad \text{Eq. S2}$$

$$D_{\mathrm{P,aver}} = C_{\mathrm{P,MEX-6f}} \cdot D_{\mathrm{MEX-6f}} + C_{\mathrm{P,MEX-6s}} \cdot D_{\mathrm{MEX-6s}} \qquad \text{Eq. S3}$$

where $D_{\mathrm{A,aver}}$ and $D_{\mathrm{P,aver}}$ are the average MEX-6 diffusion coefficients at the two sides of the cytoplasm, obtained by FRAP measurements, and $C_{\mathrm{A/P,MEX-6f}}$ and $C_{\mathrm{A/P,MEX-6s}}$ the relative concentrations of the slow and fast molecules at anterior/posterior. We used the value of $D_{\mathrm{A,aver}} = 0.55\ \mu\mathrm{m}^2/\mathrm{s}$ for the anterior (as measured in the *mNG::mex-6* strain by FRAP) and $D_{\mathrm{P,aver}} = 0.91\ \mu\mathrm{m}^2/\mathrm{s}$ at the

posterior, a value in between the experimental measurements in the *mNG::mex-6* and the *GFP::mex-6* strains. A further increase of the posterior average diffusivity $D_{\mathrm{P,aver}}$, while keeping fixed $C_{\mathrm{A,MEX-6f}}$ and $C_{\mathrm{A,MEX-6s}}$, did not allow positive solutions for $D_{\mathrm{MEX-6s}}$. We assumed that the relative concentrations of MEX-6 in the fast and slow states were similar to those of MEX-5 (see Main Text), *i.e.* $C_{\mathrm{A,MEX-6s}} = 70\%$ at the anterior and $C_{\mathrm{P,MEX-6s}} = 50\%$ at the posterior for the slow component, and $C_{\mathrm{A,MEX-6f}} = 30\%$ and $C_{\mathrm{P,MEX-6f}} = 50\%$ for the fast one (Fig. 2B, Table 1).

So the set of equations became:

$$D_{\mathrm{A,aver}} = 0.7 \cdot D_{\mathrm{MEX-6f}} + 0.3 \cdot D_{\mathrm{MEX-6s}} \qquad \text{Eq. S2}$$

$$D_{\mathrm{P,aver}} = 0.5 \cdot D_{\mathrm{MEX-6f}} + 0.5 \cdot D_{\mathrm{MEX-6s}}, \qquad \text{Eq. S3}$$

which gave D_{MEX-6f} ~1.81 μm^2/s and D_{MEX-6s} ~0.01 μm^2/s, finally converted to input velocities for the Monte Carlo using (3):

$$D_{MEX-6x} = -8 \cdot 10^{-4}\, v_{MEX-6x} + 0.125\, v_{MEX-6x}^2, \qquad x = s, f \qquad \text{Eq. S4}$$

giving v_{MEX-6f} = 3.8 μm/s for fast MEX-6 and v_{MEX-6s} = 0.29 μm/s for slow MEX-6.

Statistical Analyses

All statistical analyses were performed in GraphPad Prism 10.2.2. Details on the statistics and precise P values are reported in Table S6.

Fig. S1

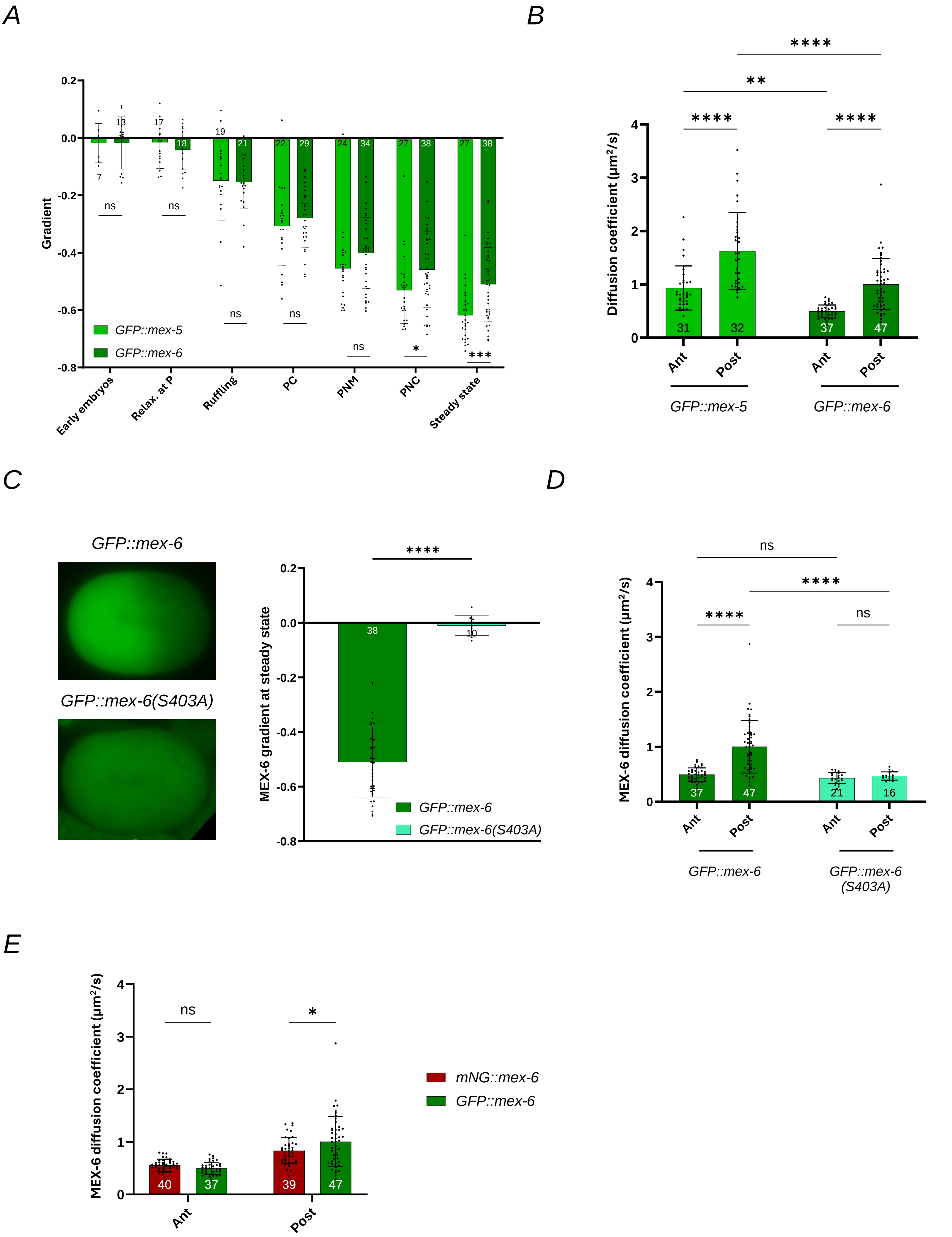
A
B
C
D
E
Gradient
0.2
0.0
-0.2
-0.4
-0.6
-0.8
ns
ns
ns
ns
ns
*
***
GFP::mex-5
GFP::mex-6
Early embryos
Relax. at P
Ruffling
PC
PNM
PNC
Steady state
****
**
****
****
Diffusion coefficient (µm²/s)
31
32
37
47
Ant
Post
Ant
Post
GFP::mex-5
GFP::mex-6
GFP::mex-6
GFP::mex-6(S403A)
MEX-6 gradient at steady state
38
10
GFP::mex-6
GFP::mex-6(S403A)
ns
****
****
ns
MEX-6 diffusion coefficient (µm²/s)
37
47
21
16
Ant
Post
Ant
Post
GFP::mex-6
GFP::mex-6
(S403A)
MEX-6 diffusion coefficient (µm²/s)
ns
*
40
37
39
47
mNG::mex-6
GFP::mex-6
Ant
Post

Figure S1. GFP::MEX-5 and GFP::MEX-6 gradients are similar but GFP::MEX-6 diffuses slower. A) GFP::MEX-5 and GFP::MEX-6 gradients as a function of time, shown for relevant timepoints during the first cell division in the one-cell embryo (see legend below). The gradient is quantified as the slope of the linear fit of the signal along the anterior-posterior axis ($\partial Intensity/\partial x$). For simplicity, it will be referred to as “Gradient” or “Protein gradient” (*e.g.* MEX-6 gradient) in all the subsequent graphs. B) Comparison of GFP::MEX-5 and GFP::MEX-6 diffusivity at both anterior and posterior sides of one-cell embryos at steady state. C) Left, images of *GFP::mex-6* and *GFP::mex-6(S403A)* one-cell embryos and, right, quantification of their gradients at steady state. D) Comparison of MEX-6 diffusivity in the *GFP::mex-6* strain and in the *GFP::mex-6(S403A)* mutant, measured at anterior and posterior sides of embryos at steady state. E) Direct comparison of the diffusion coefficient of mNG::MEX-6 and GFP::MEX-6, in both the anterior and posterior cytoplasm of one-cell embryos at steady state. The plot compares the data reported in Fig. 1E and Fig S1D.

In all the plots, the bars represent the average values of the different measurements, and the error bars the SD. The number N of analyzed embryos is reported for each condition. Embryos from different experiments were pooled together and, for A) and B), these bar plots were used as control conditions in the following experiments.

In A) and C), the statistical analysis was performed, for each stage separately, using the two-tailed unpaired t test. In B), D) and E), the two-way ANOVA test, with Tukey’s multiple comparison, was used.

Legend: Relax. at P= relaxation at posterior; PC= pseudo-cleavage; PNM= pronuclear meeting; PNC= pronuclear centration. Ant= anterior; Post= posterior.
For the statistics: ns: $p>0.5$, *: $p<0.05$, **: $p<0.01$, ***: $p<0.001$, ****: $p<0.0001$

These conventions are kept in all Supplementary Figures.

A

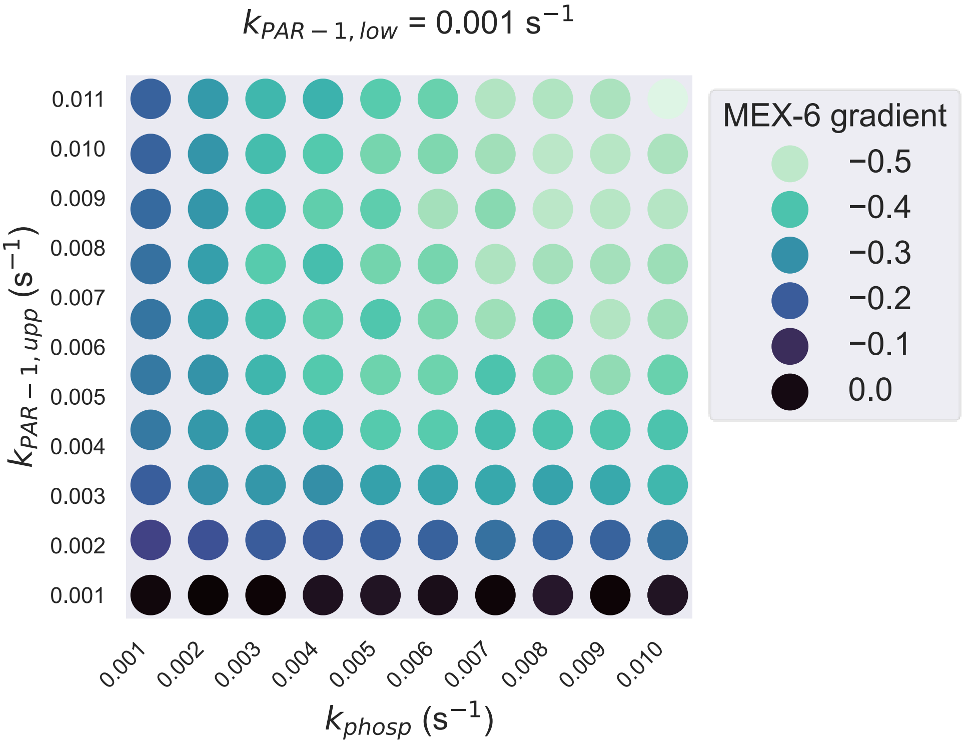

$k_{PAR-1,low}$ = 0.001 s^{-1}
MEX-6 gradient
−0.5
−0.4
−0.3
−0.2
−0.1
0.0
$k_{PAR-1,upp}$ (s^{-1})
k_{phosp} (s^{-1})

B

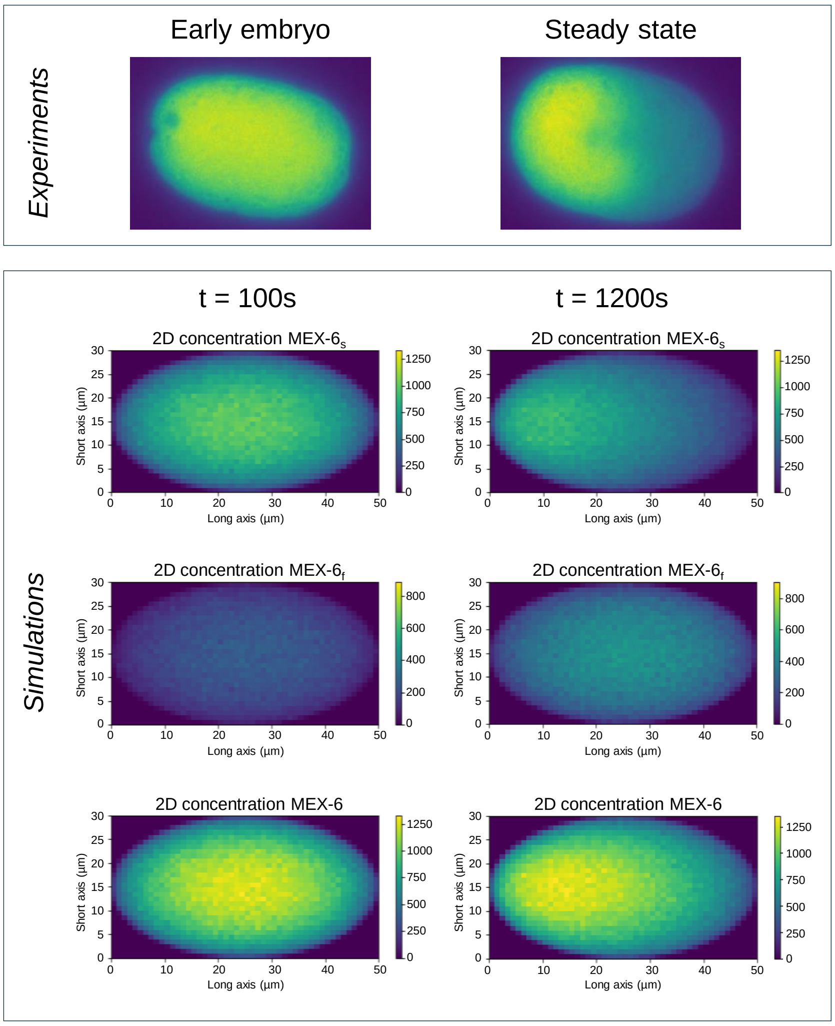

Early embryo
Steady state
Experiments
t = 100s
t = 1200s
2D concentration MEX-6$_s$
2D concentration MEX-6$_f$
2D concentration MEX-6
Short axis (µm)
Long axis (µm)
Simulations

Figure S2. Parameter sensitivity analysis for the model reaction rates and MEX-6 concentration gradient in vivo and in silico. A) Simulations of the MEX-6 steady-state gradient (color scale) for different values of the reaction rates. In these simulations, $k_{PAR-1.low}$ was fixed to 0.001 s^{-1}, while $k_{PAR-1.upp}$ and k_{phosp} were varied within a range of values centered on the parameters used for the simulation of MEX-5 gradient in (3). B) The box at the top shows the localization of MEX-6 in an early embryo (left) and at steady state (right), as detected from time-lapse microscopy. The box at the bottom shows the simulated distribution of MEX-6 after 100 s seconds from the beginning of the simulations (left) and at the steady state (20 min timepoint, right). The top row shows the distribution of the slow component of MEX-6 (MEX-6_s), the middle row shows the fast component of MEX-6 (MEX-6_f) and the bottom row the total protein concentration (MEX-6). These images were obtained by projecting onto a 2D plane the molecules contained in a central, 5-um thick slice of the 3D ellipsoidal volume representing the embryo.

A

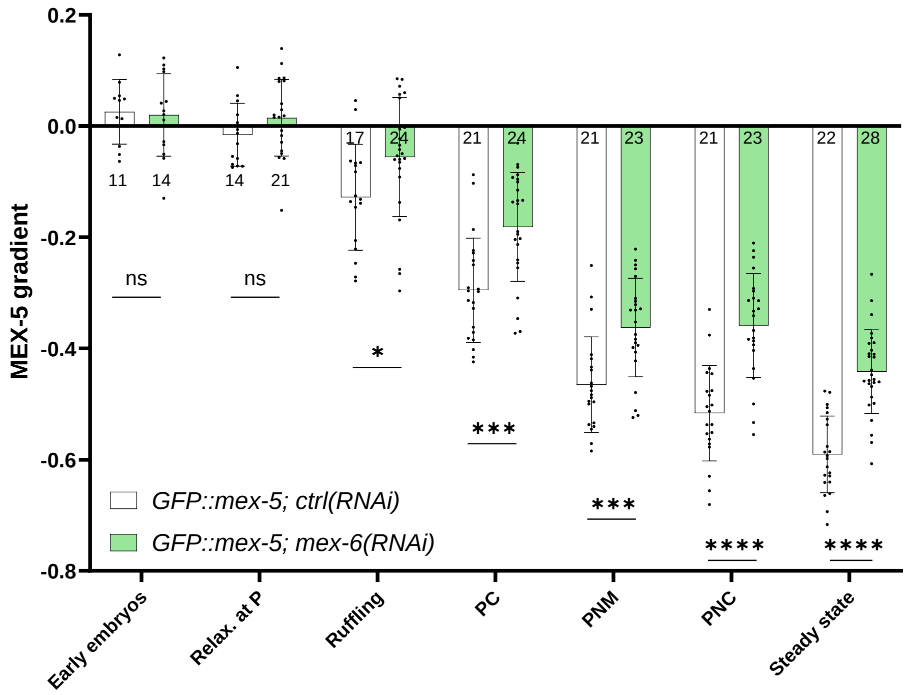
MEX-5 gradient
0.2
0.0
-0.2
-0.4
-0.6
-0.8
11
14
14
21
17
24
21
24
21
23
21
23
22
28
ns
ns
*
***
***
****
****
GFP::mex-5; ctrl(RNAi)
GFP::mex-5; mex-6(RNAi)
Early embryos
Relax. at P
Ruffling
PC
PNM
PNC
Steady state

B

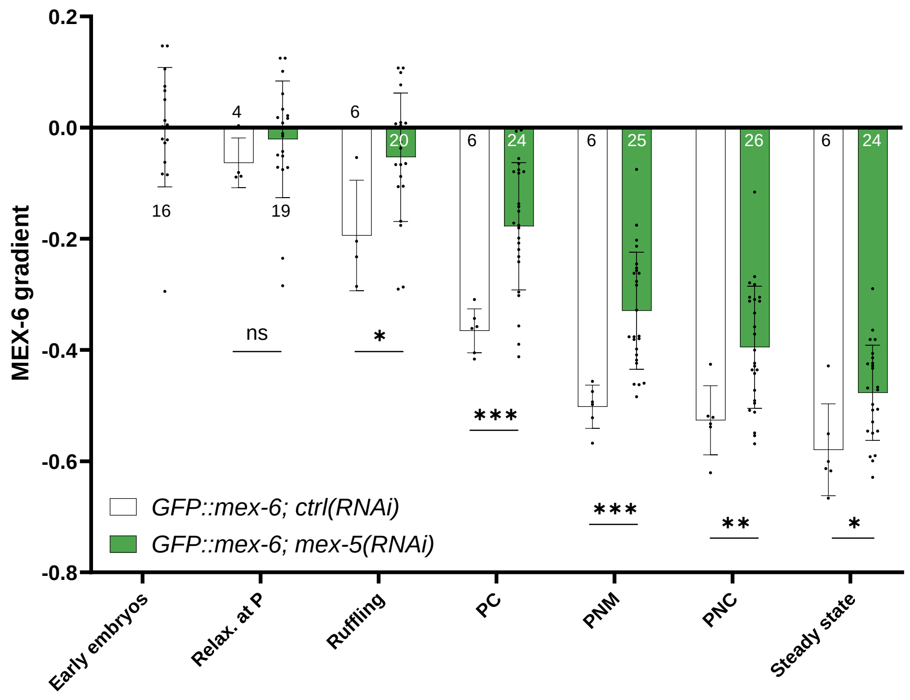
MEX-6 gradient
0.2
0.0
-0.2
-0.4
-0.6
-0.8
16
4
19
6
20
6
24
6
25
26
6
24
ns
*
***
***
**
*
GFP::mex-6; ctrl(RNAi)
GFP::mex-6; mex-5(RNAi)
Early embryos
Relax. at P
Ruffling
PC
PNM
PNC
Steady state

Figure S3. MEX-5 and MEX-6 depletions reduce the steepness of each other's gradients in the GFP-labelled strains. Comparison of the dynamics of: A) MEX-5 gradient after treatment with the *ctrl(RNAi)* and *mex-6(RNAi)* in the *GFP::mex-5* strain; B) MEX-6 gradient after treatment with *ctrl(RNAi)* and *mex-5(RNAi)* in the *GFP::mex-6* strain.

In the plots, the statistical analysis was performed, for each stage separately, using the two-tailed unpaired t test. The bars represent the average values of the different measurements and the error bars the SD. The number N of analyzed embryos is reported for each condition.

Fig. S4

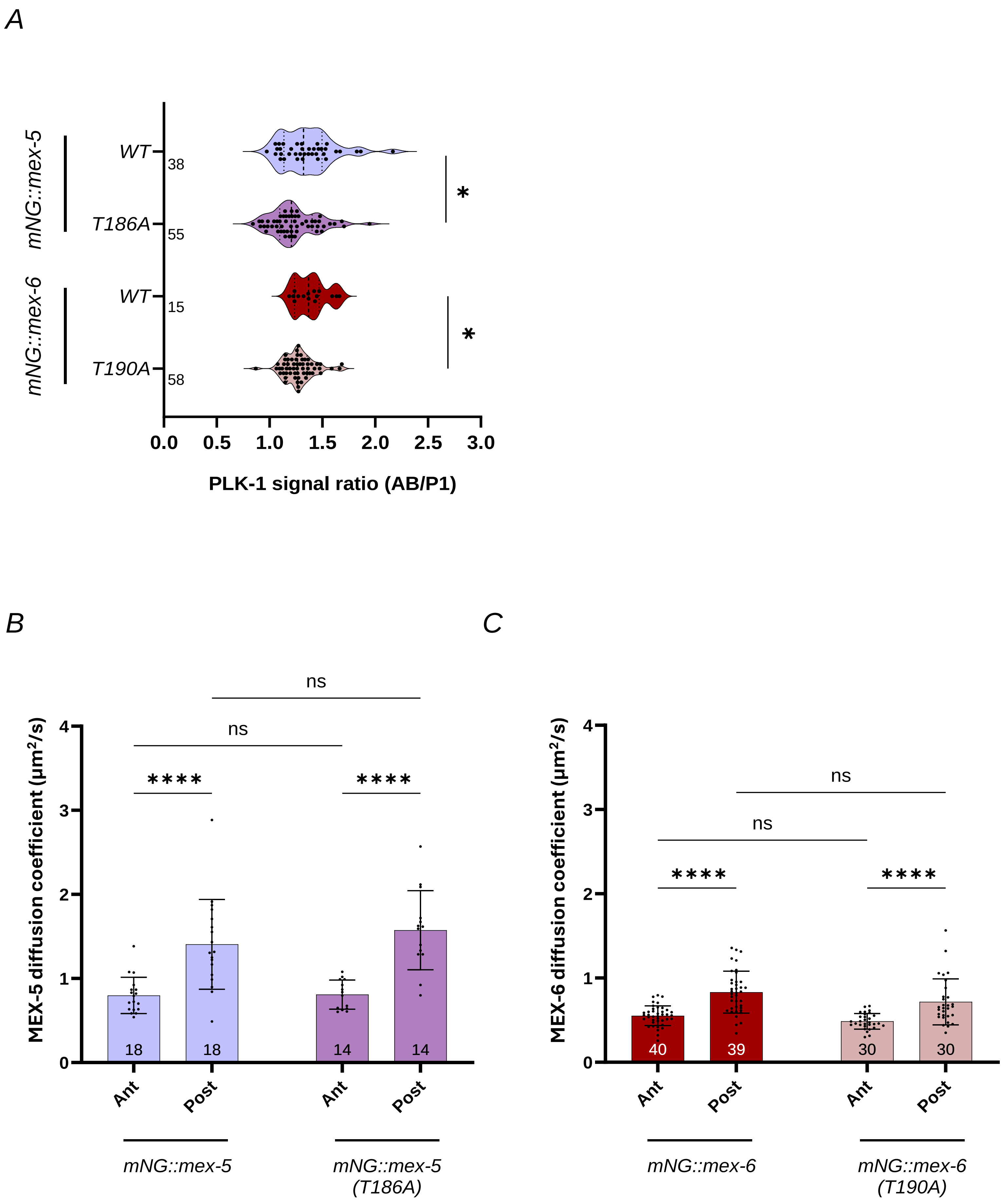
A
mNG::mex-5
mNG::mex-6
WT
T186A
WT
T190A
38
55
15
58
*
*
0.0
0.5
1.0
1.5
2.0
2.5
3.0
PLK-1 signal ratio (AB/P1)
B
C
MEX-5 diffusion coefficient (μm²/s)
ns
ns
****
****
18
18
14
14
Ant
Post
Ant
Post
mNG::mex-5
mNG::mex-5 (T186A)
MEX-6 diffusion coefficient (μm²/s)
ns
ns
****
****
40
39
30
30
Ant
Post
Ant
Post
mNG::mex-6
mNG::mex-6 (T190A)

Figure S4. The PDS mutations in MEX-5 and MEX-6 reduce the gradient of PLK-1 but do not change MEX-5 and MEX-6 diffusivity. A) Analysis of the asymmetry of the PLK-1 distribution in 2-cell embryos of the *mNG::mex-5(T186A)* and *mNG::mex-6(T190A)* strains, compared to their relative parental strains. The asymmetry was estimated as the ratio of PLK-1 signal, detected by immunofluorescence, in the anterior cell AB over the signal in the posterior P1 cell. The statistical analysis was performed using the Mann-Whitney test, separately for the *mex-5* and the *mex-6* strains. B-C) Measurement of the diffusivity of MEX-5 in *mNG::mex-5(T186A)* embryos (C) and of MEX-6 in *mNG::mex-6(T190A)* embryos (D), compared to the parental strains. The statistical analysis was performed using the two-way ANOVA test, with Tukey's multiple comparison.

In A), the violin plots report the median (dashed line) and the quartiles (dotted lines) of the distributions. In B-C), the control conditions *mNG::mex-5* and *mNG::mex-6* represent the same data as in Fig. 1C. The bars represent the average values of the different measurements and the error bars the SD. The number N of analyzed embryos is reported for each condition.

Supplementary Tables

Table S1. List of strains used in this work.

Strain Name	Genotype	Source
LP373	*mNG::mex-5*	Daniel Dickinson (11), CGC
PHX6363	*mNG::mex-6*	SunyBiotech, this study
EGD104	*GFP::mex-5*	Courtesy of Erik Griffin
PHX7226	*GFP::mex-6*	SunyBiotech, this study
ZU330 – ZU331	*mNG::mex-6(S403A), two lines*	This study
ZU335 – ZU336	*GFP::mex-6(S403A), two lines*	This study
PHX8990	*mNG::mex-5(R277E; K321E)*	SunyBiotech, this study
PHX5777	*mex-5(MEX-6 ZF)*	SunyBiotech, this study
PHX7161	*mNG::mex-5(MEX-6 ZF)*	SunyBiotech, this study
PHX9066	*mNG::mex-6(MEX-5 ZF)*	SunyBiotech, this study
JH3405	*mex-5(T186A)*	Courtesy of Geraldine Seydoux
PHX8576	*mNG::mex-5(T186A)*	SunyBiotech, this study
PHX4753	*mex-6(T190A)*	SunyBiotech, this study
PHX8102	*mNG::mex-6(T190A)*	SunyBiotech, this study
KK1273	*GFP::par-2*	Ken Kemphues, CGC
ZU370	*GFP::par-2; mex-5(T186A)*	This study
ZU371	*mex-6(T190A); GFP::par-2*	This study
KK1262	*par-1::GFP::par-1 exon11a*	Diane Morton, CGC
ZU375	*mex-6(T190A); par-1::GFP*	This study
ZU376	*mex-5(T186A); par-1::GFP*	This study

Table S2. List of oligos used in this work to produce CRISPR strains.

CRISPR strain	Sequence	Function
mNG::mex-6, PHX6363	ctcaacagtgaatttttaagacagaaaATGGTCTCCAAGGGAG<br>AGGAGGACAACATGGCCTCCCTCCCAGCCACCCA<br>CGAGCTCCACATCTTCGGATCCATCAACGGAGTCG<br>ACTTCGACATGGTCGGACAAGGAACCGGAAACCC<br>AAACGACGGATACGAGGAGCTCAACCTCAAGTCCA<br>CCAAGgtaagtttaaacatatatatactaactaaccctgattatttaaatttt<br>cagGGAGACCTCCAATTCTCCCCATGGATCCTCGTC<br>CCACACATCGGATACGGATTCCACCAATACCTCCCA<br>TACCCAGACGGAATGTCCCCATTCCAAGCCGCCAT<br>GGTCGACGGATCCGGATACCAAGTCCACCGTACCA<br>TGCAATTCGAGGACGGAGCCTCCCTCACCGTCAAC<br>TACCGTTACACCTACGAGGGATCCCACATCAAGgtaa<br>gtttaaacagttcggtactaactaaccatacatatttaaattttcagGGAG<br>AGGCCCAAGTCAAGGGAACCGGATTCCCAGCCGA<br>CGGACCAGTCATGACCAACTCCCTCACCGCCGCC<br>GACTGGTGCCGTTCCAAGAAGACCTACCCAAACGA<br>CAAGgtaagtttaaacatgattttactaactaactaatctgatttaaattttca<br>gACCATCATCTCCACCTTCAAGTGGTCCTACACCAC<br>CGGAAACGGAAAGCGTTACCGTTCCACCGCCCGT<br>ACCACCTACACCTTCGCCAAGCCAATGGCCGCCAA<br>CTACCTCAAGAACCAACCAATGTACGTCTTCCGTAA<br>GACCGAGCTCAAGCACTCCAAGACCGAGCTCAAC<br>TTCAAGGAGTGGCAAAAGGCCTTCACCGACGTCAT<br>GGGAATGGACGAGCTCTACAAGGGAGGTTCTGGA<br>ACAGCAACATCCAATAGTGCTCCTTTGGCCGGTGG<br>ATCATTGTCTTCTT	Modified endogenous sequence
	TGTAGACTGTACCTGGTGGG	PCR and sequencing forward oligo
	ACTCATGGTATCGTCGTTG	PCR and sequencing reverse oligo
	TCGACTATTCCTCAACAGTG	Sequencing forward oligo - 2
GFP::mex-6, PHX7226	caacagtgaatttttaagacagaaaATGAGTAAAGGAGAAGAA<br>CTTTTCACTGGAGTTGTCCCAATTCTTGTTGAATTA<br>GATGGTGATGTTAATGGGCACAAATTTTCTGTCAG<br>TGGAGAGGGTGAAGGTGATGCAACATACGGAAAA<br>CTTACCCTTAAATTTATTTGCACTACTGGAAAACTA<br>CCTGTTCCATGGgtaagtttaaacatatatatactaactaaccctga<br>ttatttaaattttcagCCAACACTTGTCACTACTTTCTgTTAT<br>GGTGTTCAATGCTTcTCgAGATACCCAGATCATATG<br>AAACgGCATGACTTTTTCAAGAGTGCCATGCCCGA<br>AGGTTATGTACAGGAAAGAACTATATTTTTCAAAGA<br>TGACGGGAACTACAAGACACgtaagtttaaacagttcggtact<br>aactaaccatacatatttaaattttcagGTGCTGAAGTCAAGTTT<br>GAAGGTGATACCCTTGTTAATAGAATCGAGTTAAAA<br>GGTATTGATTTTAAAGAAGATGGAAACATTCTTGGA<br>CACAAATTGGAATACAACTATAACTCACACAATGTA	Modified endogenous sequence

	TACATCATGGCAGACAAACAAAAGAATGGAATCAAAGTTgtaagtttaaacatgattttactaactaactaatctgatttaaattttcagAACTTCAAAATTAGACACAACATTGAAGATGGAAGCGTTCAACTAGCAGACCATTATCAACAAAATACTCCAATTGGCGATGGCCCTGTCCTTTTACCAGACAACCATTACCTGTCCACACAATCTGCCCTTTCGAAAGATCCCAACGAAAAGAGAGACCACATGGTCCTTCTTGAGTTTGTAACAGCTGCTGGGATTACACATGGCATGGATGAACTATACAAAGGAGGTTCTGGAACAGCAACATCCAATAGTGCTCCTTTGGCCGGTGGATCATTGTCTTCTT	
	TATTTGTCTTTGTCGAGGCG	PCR forward oligo
	ATGGAAGTACATTGGAGCAG	PCR reverse oligo
	CGATTCTATTAACAAGGGTAT	Sequencing forward oligo
	TGCCCGAAGGTTATGTACAG	Sequencing reverse oligo
mNG::mex-6(S403A), two lines,<br>ZU330 – ZU331<br><br>*GFP::mex-6(S403A), two lines,*<br>ZU335 – ZU336	caaatgactgttcttcaataccttcattttcagTGCCATGATGTGTTTATCAAACACGGGACGCGATTTAGAGGCAGGAGGTGATTTCAACCATCC	Modified endogenous sequence
	aaaggctacgactccaacg	PCR and sequencing forward oligo
	TGAAAAAAAGCTGAGACCGG	PCR and sequencing reverse oligo
mNG::mex-6(R277E; K321E),<br>PHX8990	CCAGAAGTTGACAGCCAACTGCCACACAACTTCAAAACTGAGCTTTGCATGACGCATGCTGCTGGAATTAATCCTTGTGCATTGGGTGCTCGGTGCAAGTTTGCTCATGGGCTCAAAGAGCTCAGAGCTTCTGATATTgtgagtttaattccattttatctcatagaattcaaaatgtcgttttcagCCGACTCGGTACCCGAACAATAAATACAAAACTGAGCTCTGCAAGAATTTTGCGCGTGGAGGATCTGGAGTTTGTCCATATGGGCTTCGATGCGAGTTTGTTCAC	Modified endogenous sequence
	GCGTACAGTTCCGTTGACTT	PCR and sequencing forward oligo
	AATCCGTCTCAAGTGTGGTG	PCR and sequencing reverse oligo
mex-5(MEX-6 ZF),<br>PHX5777	ATTCCAGAAACTGACAGTCAACAACCACCCAACTTTAAGACTCGTCTTTGCATGACGCACGCAGCTGGAATTAATCCTTGTGCATTGGGTGCTCGGTGCAAGTTTGCTCATGGGCTCAAAGAGCTCAGAGCTACTGATGCTCCGGCTCGCTATCCGAACAACAAATACAAAACTAAATTGTGCAAGAATTTTGCGCGTGGAGGATCTGGAGTTTGCCCATATGGGCTTCGATGCGAGTTTGTTCACCCATCGGATAAGGAATTCCAGAATATTCCTCCATATgtg	Modified endogenous sequence

	GAACAAATGGGTGTGATGCC	PCR and sequencing forward oligo
	TGCGATTCCGTCTCAAGT	PCR and sequencing reverse oligo
mNG::mex-5(MEX-6 ZF), PHX7161	taatcaattgaatgtttcagacagagaATGGTCTCCAAGGGAGAGGAGGACAACATGGCCTCCCTCCCAGCCACCCACGAGCTCCACATCTTCGGATCCATCAACGGAGTCGACTTCGACATGGTCGGACAAGGAACCGGAAACCCAAACGACGGATACGAGGAGCTCAACCTCAAGTCCACCAAGgtaagtttaaacatatatatactaactaaccctgattatttaaattttcagGGAGACCTCCAATTCTCCCCATGGATCCTCGTCCCACACATCGGATACGGATTCCACCAATACCTCCCATACCCAGACGGAATGTCCCCATTCCAAGCCGCCATGGTCGACGGATCCGGATACCAAGTCCACCGTACCATGCAATTCGAGGACGGAGCCTCCCTCACCGTCAACTACCGTTACACCTACGAGGGATCCCACATCAAGgtaagtttaaacagttcggtactaactaaccatacatatttaaattttcagGGAGAGGCCCAAGTCAAGGGAACCGGATTCCCAGCCGACGGACCAGTCATGACCAACTCCCTCACCGCCGCCGACTGGTGCCGTTCCAAGAAGACCTACCCAAACGACAAGgtaagtttaaacatgattttactaactaactaatctgatttaaattttcagACCATCATCTCCACCTTCAAGTGGTCCTACACCACCGGAAACGGAAAGCGTTACCGTTCCACCGCCCGTACCACCTACACCTTCGCCAAGCCAATGGCCGCCAACTACCTCAAGAACCAACCAATGTACGTCTTCCGTAAGACCGAGCTCAAGCACTCCAAGACCGAGCTCAACTTCAAGGAGTGGCAAAAGGCCTTCACCGACGTCATGGGAATGGACGAGCTCTACAAGGATTATAAAGACGATGACGATAAGCGTGACTACAAGGACGACGACGACAAGCGTGATTACAAGGATGACGATGACAAGAGAAAAGCGGCATCAAATAGTGTCTCGTCTTGCTGGAGGATCAGTGTCACCTACGACAA	Modified endogenous sequence
	GTTCCGAACCTCAGGTAAGG	PCR forward oligo
	TTGAAGATCGCGACCAGCAT	PCR reverse oligo
	TTACCGCGTATACCGTACTC	Sequencing forward oligo
	CTTGCATAGGATGGAAGTAC	Sequencing reverse oligo
mNG::mex-6(MEX-5 ZF), PHX9066	ATGGTCTCCAAGGGAGAGGAGGACAACATGGCCTCCCTCCCAGCCACCCACGAGCTCCACATCTTCGGATCCATCAACGGAGTCGACTTCGACATGGTCGGACAAGGAACCGGAAACCCAAACGACGGATACGAGGAGCTCAACCTCAAGTCCACCAAGgtaagtttaaacatatatatactaactaaccctgattatttaaattttcagGGAGACCTCCAATTCTCCCCATGGATCCTCGTCCCACACATCGGATACGGATTCCACCAATACCTCCCATACCCAGACGGAATGTCCCCATTCCAAGCCGCCATGGTCGACGGATCCGGATACCAAGTCCACCGTACCATGCAATTCGAGGACGGAGCCTCCCTCACCGTCAACTACCGTTACACCTACGAGGGATCCCACATCAAGgtaagtttaaacagttcggtactaactaac	Modified endogenous sequence

	catacatatttaaattttcagGGAGAGGCCCAAGTCAAGGGA<br>ACCGGATTCCCAGCCGACGGACCAGTCATGACCA<br>ACTCCCTCACCGCCGCCGACTGGTGCCGTTCCAA<br>GAAGACCTACCCAAACGACAAGgtaagtttaaacatgatttta<br>ctaactaactaatctgatttaaattttcagACCATCATCTCCACCTT<br>CAAGTGGTCCTACACCACCGGAAACGGAAAGCGTT<br>ACCGTTCCACCGCCCGTACCACCTACACCTTCGCC<br>AAGCCAATGGCCGCCAACTACCTCAAGAACCAACC<br>AATGTACGTCTTCCGTAAGACCGAGCTCAAGCACT<br>CCAAGACCGAGCTCAACTTCAAGGAGTGGCAAAA<br>GGCCTTCACCGACGTCATGGGAATGGACGAGCTC<br>TACAAGGGAGGTTCTGGAACAGCAACATCCAATAG<br>TGCTCCTTTGGCCGGTGGATCATTGTCTTCTTCAGC<br>AACGGCACAGCCACCACAACCACCTCCAGGACAT<br>CAACAGCAGCATCCACTTCCTCAAATCTACGATTCG<br>CAGgtttttaaccgtgaaacttattatttatttttcaaaataaattacagATG<br>CAATACTACTACGGATCTTCCATGCCCAATCAGCCA<br>ATTCCAACGTACACGGCACAAAACGGAGCACCTCA<br>ACAGTTCGGCACACCACCGTACTATCAAGATGCAA<br>ACGGACAATTTGGACAGGTTCCCGCTCAACAGCAA<br>ATGATGACTGCTGGTCATCCATACTTTTATATGGCCC<br>AGCCGCAGCAAGGAGGACAGCACGTTGCACAATC<br>TGGACAACCGCAGATATTTTATTATCAGCAACCATT<br>GGGTCAAATGGCTCAACAAGCTGCTCCAATGTACT<br>TCCATCCAATGCAAGCTGCTTCTACTCCTATGCTTT<br>CTGAACAAATGAGTATGATGCCACAAATCCAGTCGA<br>CTAATCCTCAACAGTCTGAACAACTGAGAAAATCGG<br>GTGCTCAAATAAGCACCACGCGTACAGTTCCGTTG<br>ACTTCGTCGACTCCGCTCCCAACAAGTCGCGAATA<br>CGAGACTGTCCAACGCGATCGAAACAGGAATAGCC<br>AATCAAGATATCAATGgtaaaattagatttgtttaataagcgttttatt<br>aatcattcttgattttccagTCCTATTGAACATGACGATCTTCC<br>CATCGATGAGATCTCGAAGATCACCATCGACAATCA<br>CAACGACGATACCATGAGTGCTGAGAAGGAAAATC<br>GTTTCAATAAGgttgctctttttcccttcaagaattacatgattttttcag<br>AATCGTGTTGAGAAATTGGGTCGCCGTGGGTTCGC<br>CAAACCAGAAGTTGACAGCCAACTGCCACACAACT<br>ACAAGACTCGTCTTTGCATGATGCACGCATCTGGA<br>ATCAAACCATGTGATATGGGTGCTCGATGCAAGTTT<br>GCTCATGGGCTCAAAGAGCTCAGAGCTTCTGATAT<br>TgtgagtttaattccattttatctcatagaattcaaaatgtcgttttcagCCGA<br>CTCGGTACCCGAACAATAAGTACAAGACAAAGCTG<br>TGCAAGAACTTTGCGCGTGGAGGAACTGGATTCTG<br>CCCGTATGGACTTCGTTGCGAGTTTGTTCATCCAAC<br>GGACACGGAATTCCAGAATATTCCTCCATATgta	
	GCGTACAGTTCCGTTGACTT	PCR and sequencing forward oligo
	AATCCGTCTCAAGTGTGGTG	PCR and sequencing reverse oligo
mNG::mex-5(T186A), PHX8576	CACACGCACAGCTCCATTGACCTCGAGTGCGCCTT<br>TGCCAACAAGTCTCGAATACGAGACTG	Modified endogenous sequence
	ACCAGCCAATGGCGACGTAT	PCR and sequencing forward oligo

	AACGAAGTCCATACGGGCAG	PCR and sequencing reverse oligo
mex-6(T190A), PHX4753 *mNG::mex-6(T190A)*, PHX8102	CACGCGTACAGTTCCGTTGACTTCGAGTGCGCCGTTGCCAACAAGTCGCGAATACGAGACTG	Modified endogenous sequence
	AGCCAATTCCAACGTACACG	PCR and sequencing forward oligo
	AGCTCTTTGAGCCCATGAGC	PCR and sequencing reverse oligo

In green, the sequence of the fluorescence tags; in purple, the sequence of the used linkers; in orange, the swapped ZF domains; in blue, the sequences of the synonymous mutations; in red the introduced mutations.

Table S3. Quantification of the diffusivity measurements performed in the study. Averages diffusion coefficients are reported with their standard deviations.

Figure	Strain	Embryo side	Average D_c ($\mu m^2/s$)	SD D_c ($\mu m^2/s$)
1C	*mNG::mex-5*	Anterior	0.80	0.21
		Posterior	1.41	0.53
	mNG::mex-6	Anterior	0.55	0.12
		Posterior	0.83	0.25
1E	*mNG::mex-6(S403A)*	Anterior	0.51	0.14
		Posterior	0.54	0.22
	mNG::mex-6(R277E; K321E)	Anterior	1.14	0.30
		Posterior	1.65	0.69
3C	*mNG::mex-5(MEX-6 ZF)*	Anterior	1.02	0.25
		Posterior	1.64	0.66
	mNG::mex-6(MEX-5 ZF)	Anterior	0.55	0.11
		Posterior	0.91	0.27
S1B	*GFP::mex-5*	Anterior	0.93	0.41
		Posterior	1.63	0.72
	GFP::mex-6	Anterior	0.49	0.12
		Posterior	1.00	0.48
S1D	*GFP::mex-6(S403A)*	Anterior	0.43	0.10
		Posterior	0.47	0.08
S4B	*mNG::mex-5(T186A)*	Anterior	0.81	0.17
		Posterior	1.57	0.47
S4C	*mNG::mex-6(T190A)*	Anterior	0.49	0.09
		Posterior	0.72	0.27

Table S4. List of oligos used to mutagenize and clone the protein sequences.

Plasmid	Primers (5'→3')	Function
oMG1966<br><br><br><br>oMG1967	Forward:<br>GGGGACAAGTTTGTACAAAAAAGCAGGCTTGATGAAAGCGGCATCAAATAGTG<br><br>Reverse:<br>GGGGACCACTTTGTACAAGAAAGCTGGGTGCTAATAGTGTCCTCTTAGTCCG	Forward and reverse primers with Gateway overhang to amplify full length of MEX-5 .
oMG1962<br><br><br><br>oMG1963	Forward:<br>GGGGACAAGTTTGTACAAAAAAGCAGGCTTGATGACAGCAACATCCAATAGTG<br><br>Reverse:<br>GGGGACCACTTTGTACAAGAAAGCTGGGTGCTAATAGTGTCCTCGCAGACC	Forward and reverse primers with Gateway overhang to amplify full length of MEX-6.
oMG1225<br><br><br><br>oMG1226	Forward:<br>CTCCATTGACCTCGTCTGCTCCTCTTCCAACAAGT<br><br>Reverse:<br>ACTTGTTGGAAGAGGAGCAGACGAGGTCAATGGAG	Forward and reverse primers for mutagenesis of threonine T186 to alanine in MEX-5.
oMG1978<br><br><br>oMG1979	Forward:<br>TTCGTCGGCTCCGCTCCC<br><br>Reverse:<br>GGGAGCGGAGCCGACGAA	Forward and reverse primers for mutagenesis of threonine T190 to alanine in MEX-6.

In red, the Gateway overhangs. In green, the nucleotides added to keep the ORF. In blue, the nucleotide replacements to obtain the polo-docking site T to A mutations.

Table S5. List of the plasmids used in the yeast two-hybrid experiments.

Plasmid	Backbone	Insert	Tag	Reference
-	pDONR201	Empty	-	Invitrogen
-	pDEST22	Empty	Y2H AD	Invitrogen
-	pDEST32	Empty	Y2H DBD	Invitrogen
pMG1495	pDONR201	MEX-5	-	This study
pMG1502	pDEST32	MEX-5	Y2H DBD	This study
pMG1494	pDONR201	MEX-6	-	This study
pMG1508	pDEST22	MEX-6	Y2H AD	This study
pMG891	pDONR201	MEX-5(T186A)	-	This study
pMG1631	pDEST32	MEX-5(T186A)	Y2H DBD	This study
pMG1632	pDONR201	MEX-6(T190A)	-	This study
pMG1633	pDEST22	MEX-6(T190A)	Y2H AD	This study

Table S6. Details on the statistical tests performed in all the main and supplementary Figures.

**Figure 1B**	*mNG::mex-5* vs *mNG::mex-6*, per stage		
**Stage**	**P**	**DF**	**t**
Early embryos	0.587	15	0.169
Relaxation at posterior	0.150	42	1.468
Ruffling	0.988	66	0.015
Pseudo-cleavage	0.885	81	0.146
Pronuclear meeting	0.456	85	0.749
Pronuclear centration	0.714	87	0.367
Steady state	0.606	91	0.517
**Figure 1C**	*mNG::mex-5* vs *mNG::mex-6*		
	**F (DFn, DFd)**	**P**	
Interaction	F (1, 111) = 8.677	0.0039	
Row factor	F (1, 111) = 63.20	<0.0001	
Column factor	F (1, 111) = 54.08	<0.0001	
**Tukey's test**	**Predicted (LS) mean diff.**	**95% CI of diff.**	**Adjusted P**
Ant: mNG::mex-6 vs. Ant:mNG::mex-5	-0.246	-0.4513 to -0.04054	0.012
Ant: mNG::mex-6 vs. Post:mNG::mex-6	-0.279	-0.4420 to -0.1163	0.0001
Ant: mNG::mex-6 vs. Post:mNG::mex-5	-0.854	-1.059 to -0.6484	<0.0001
Ant: mNG::mex-5 vs. Post:mNG::mex-6	-0.033	-0.2394 to 0.1729	0.975
Ant: mNG::mex-5 vs. Post:mNG::mex-5	-0.608	-0.8490 to -0.3666	<0.0001
Post:mNG::mex-6 vs. Post:mNG::mex-5	-0.575	-0.7808 to -0.3684	<0.0001
**Figure 1D**	*mNG::mex-6* vs *mNG::mex-6(S403A)*		
**Stage**	**P**	**DF**	**t**
Steady state	<0.000001	57	19.62
**Figure 1E**	*mNG::mex-6* vs *mNG::mex-6(S403A)* vs *mNG::mex-6(R277E; K321E)*		
	**F (DFn, DFd)**	**P**	
Interaction	F (2, 153) = 7.868	0.0006	
Row factor	F (1, 153) = 33.77	<0.0001	
Column factor	F (2, 153) = 90.01	<0.0001	
**Tukey's test**	**Predicted (LS) mean diff.**	**95% CI of diff.**	**Adjusted P**
Ant: mNG::mex-6 vs. Ant: mNG::mex-6(S403A)	0.04164	-0.1312 to 0.2145	0.9822
Ant: mNG::mex-6 vs. Ant: mNG::mex-6(R277E; K321E)	-0.5837	-0.8339 to -0.3335	<0.0001
Ant: mNG::mex-6 vs. Post:mNG::mex-6	-0.2792	-0.4446 to -0.1138	<0.0001
Ant: mNG::mex-6 vs. Post: mNG::mex-6(S403A)	0.008129	-0.1770 to 0.1933	>0.9999

Ant: mNG::mex-6 vs. Post: mNG::mex-6(R277E; K321E)	-1.095	-1.355 to -0.8354	<0.0001
Ant: mNG::mex-6(S403A) vs. Ant: mNG::mex-6(R277E; K321E)	-0.6253	-0.8812 to -0.3694	<0.0001
Ant: mNG::mex-6(S403A) vs. Post: mNG::mex-6	-0.3208	-0.4946 to -0.1470	<0.0001
Ant: mNG::mex-6(S403A) vs. Post: mNG::mex-6(S403A)	-0.03351	-0.2262 to 0.1592	0.996
Ant: mNG::mex-6(S403A) vs. Post: mNG::mex-6(R277E; K321E)	-1.137	-1.402 to -0.8716	<0.0001
Ant: mNG::mex-6(R277E; K321E) vs. Post: mNG::mex-6	0.3045	0.05359 to 0.5554	0.0078
Ant: mNG::mex-6(R277E; K321E) vs. Post: mNG::mex-6(S403A)	0.5918	0.3275 to 0.8561	<0.0001
Ant: mNG::mex-6(R277E; K321E) vs. Post: mNG::mex-6(R277E; K321E)	-0.5116	-0.8327 to -0.1905	0.0001
Post: mNG::mex-6 vs. Post: mNG::mex-6(S403A)	0.2873	0.1012 to 0.4734	0.0002
Post: mNG::mex-6 vs. Post: mNG::mex-6(R277E; K321E)	-0.8161	-1.077 to -0.5556	<0.0001
Post: mNG::mex-6(S403A) vs. Post: mNG::mex-6(R277E; K321E)	-1.103	-1.377 to -0.8299	<0.0001
**Figure 1F**	*mNG::mex-6* vs *mNG::mex-6(R277E; K321E)*, per stage		
**Stage**	**P**	**DF**	**t**
Early embryos	0.589	22	0.548
Relaxation at posterior	0.341	42	0.963
Ruffling	0.0007	59	3.581
Pseudo-cleavage	0.00001	75	4.657
Pronuclear meeting	0.00004	78	4.37
Pronuclear centration	<0.000001	79	6.364
Steady state	<0.000001	86	10.64
**Figure 3C**	*mNG::mex-5 vs mNG::mex-5(MEX-6 ZF)*		
	**F (DFn, DFd)**	**P**	
Interaction	F (1, 94) = 0.007499	0.9312	
Row factor	F (1, 94) = 40.55	<0.0001	
Column factor	F (1, 94) = 5.412	0.0221	
**Tukey's test**	**Predicted (LS) mean diff.**	**95% CI of diff.**	**Adjusted P**
Ant: mNG::mex-5 vs. Ant: mNG::mex-5(MEX-6 ZF)	-0.2167	-0.5704 to 0.1369	0.382
Ant: mNG::mex-5 vs. Post:mNG::mex-5	-0.6078	-1.010 to -0.2055	0.0009
Ant: mNG::mex-5 vs. Post: mNG::mex-5(MEX-6 ZF)	-0.8413	-1.204 to -0.4791	<0.0001
Ant: mNG::mex-5(MEX-6 ZF) vs. Post: mNG::mex-5	-0.3911	-0.7448 to -0.03741	0.0241
Ant: mNG::mex-5(MEX-6 ZF) vs. Post: mNG::mex-5(MEX-6 ZF)	-0.6246	-0.9318 to -0.3174	<0.0001
Post: mNG::mex-5 vs. Post: mNG::mex-5(MEX-6 ZF)	-0.2335	-0.5957 to 0.1287	0.3366
**Figure 3C**	*mNG::mex-6* vs *mNG::mex-6(MEX-5 ZF)*		
	**F (DFn, DFd)**	**P**	
Interaction	F (1, 114) = 1.275	P=0.2612	

Row factor	F (1, 114) = 70.87	P<0.0001	
Column factor	F (1, 114) = 0.8817	P=0.3497	
**Tukey's test**	**Predicted (LS) mean diff.**	**95% CI of diff.**	**Adjusted P**
Ant:mNG::mex-6 vs. Ant:mNG::mex-6(MEX-5 ZF)	0.007284	-0.1299 to 0.1445	0.999
Ant:mNG::mex-6 vs. Post:mNG::mex-6	-0.2792	-0.3938 to -0.1646	<0.0001
Ant:mNG::mex-6 vs. Post:mNG::mex-6(MEX-5 ZF)	-0.3584	-0.5029 to -0.2138	<0.0001
Ant:mNG::mex-6(MEX-5 ZF) vs. Post:mNG::mex-6	-0.2865	-0.4243 to -0.1486	<0.0001
Ant:mNG::mex-6(MEX-5 ZF) vs. Post:mNG::mex-6(MEX-5 ZF)	-0.3657	-0.5292 to -0.2021	<0.0001
Post:mNG::mex-6 vs. Post:mNG::mex-6(MEX-5 ZF)	-0.07921	-0.2243 to 0.06590	0.4875
**Figure 3D**	*mNG::mex-5* vs *mNG::mex-5(MEX-6 ZF)*, per stage		
**Stage**	**P**	**DF**	**t**
Early embryos	0.401	14	0.865
Relaxation at posterior	0.959	33	0.052
Ruffling	0.952	53	0.06
Pseudo-cleavage	0.194	60	1.313
Pronuclear meeting	0.0007	65	3.583
Pronuclear centration	0.00004	69	4.398
Steady state	0.00002	72	4.643
**Figure 3E**	*mNG::mex-6* vs *mNG::mex-6(MEX-5 ZF)*, per stage		
**Stage**	**P**	**DF**	**t**
Early embryos	0.198973	18	1.334
Relaxation at posterior	0.802542	39	0.252
Ruffling	0.501865	55	0.676
Pseudo-cleavage	0.17381	70	1.374
Pronuclear meeting	0.230361	76	1.209
Pronuclear centration	0.066736	78	1.859
Steady state	0.15227	81	1.445
**Figure 4A**	*mNG::mex-5; ctrl(RNAi)* vs *mNG::mex-5; mex-6(RNAi)*, per stage		
**Stage**	**P**	**DF**	**t**
Early embryos	0.424	28	0.812
Relaxation at posterior	0.598	35	0.533
Ruffling	0.765	43	0.301
Pseudo-cleavage	0.5	47	0.68
Pronuclear meeting	0.369	49	0.907
Pronuclear centration	0.196	49	1.312
Steady state	0.0002	53	3.971

**Figure 4B**	*mNG::mex-6; ctrl(RNAi)* vs *mNG::mex-6; mex-5(RNAi)*, per stage		
**Stage**	**P**	**DF**	**t**
Early embryos	0.057	25	0.576
Relaxation at posterior	0.33	40	0.982
Ruffling	0.0012	43	3.465
Pseudo-cleavage	0.00013	49	4.15
Pronuclear meeting	0.00003	48	4.609
Pronuclear centration	0.00007	51	4.327
Steady state	0.00139	55	3.367
**Figure 4C**	*mNG::mex-5* vs *mNG::mex-5(T186A)*, per stage		
**Stage**	**P**	**DF**	**t**
Early embryos	0.563	11	0.6
Relaxation at posterior	0.052	33	2.012
Ruffling	0.626	48	0.49
Pseudo-cleavage	0.95	53	0.062
Pronuclear meeting	0.523	57	0.642
Pronuclear centration	0.235	63	1.2
Steady state	0.008	64	2.732
**Figure 4D**	*mNG::mex-6* vs *mNG::mex-6(T190A)*, per stage		
**Stage**	**P**	**DF**	**t**
Early embryos	0.94	24	0.076
Relaxation at posterior	0.366	46	0.913
Ruffling	0.049	64	2.007
Pseudo-cleavage	0.003	80	3.014
Pronuclear meeting	0.018	83	2.407
Pronuclear centration	0.028	84	2.231
Steady state	0.074	88	1.807
**Figure 5A**			
	**Kruskal-Wallis statistic**	**P**	
	25.33	P<0.0001	
**Dunn's multiple comparisons test**	**Mean rank diff.**	**Adjusted P**	
GFP::par-2 vs. GFP::par-2; mex-5(T186A)	-29.87	0.0001	
GFP::par-2 vs. mex-6(T190A); GFP::par-2	11.18	0.5016	
GFP::par-2; mex-5(T186A) vs. mex-6(T190A); GFP::par-2	41.05	<0.0001	
**Figure 5B**			
	**Kruskal-Wallis statistic**	**P**	
	12.46	0.0020	
**Dunn's multiple comparisons test**	**Mean rank diff.**	**Adjusted P Value**	
mex-6(T190A); par-1::GFP vs.	-12.05	0.5004	

mex-5(T186A); par-1::GFP			
mex-6(T190A); par-1::GFP vs. par-1::GFP	15.57	0.3077	
mex-5(T186A); par-1::GFP vs. par-1::GFP	27.62	0.0013	
**Figure 5C**			
	**F (DFn, DFd)**	**P**	
	F (2, 138) = 2.338	0.1003	
**Tukey's multiple comparisons test**	**Mean Diff.**	**95.00% CI of diff.**	**Adjusted P Value**
par-1::GFP vs. mex-5(T186A); par-1::GFP	0.03312	-0.003491 to 0.06974	0.0850
par-1::GFP vs. mex-6(T190A); par-1::GFP	0.01523	-0.02888 to 0.05935	0.6924
mex-5(T186A); par-1::GFP vs. mex-6(T190A); par-1::GFP	-0.01789	-0.05885 to 0.02307	0.5563
**Figure S1A**	*GFP::mex-5* vs *GFP::mex-6*, per stage		
**Stage**	**P**	**DF**	**t**
Early embryos	0.990258	18	0.012
Relaxation at posterior	0.349	33	0.949
Ruffling	0.902	38	0.124
Pseudo-cleavage	0.411	49	0.828
Pronuclear meeting	0.116	56	1.597
Pronuclear centration	0.027	63	2.264
Steady state	0.0004	63	3.736
**Figure S1B**	*GFP::mex-5* vs *GFP::mex-6*		
	**F (DFn, DFd)**	**P**	
Interaction	F (1, 143) = 1.346	0.248	
Row factor	F (1, 143) = 57.41	<0.0001	
Column factor	F (1, 143) = 44.81	<0.0001	
**Tukey's test**	**Predicted (LS) mean diff.**	**95% CI of diff.**	**Adjusted P**
Ant:GFP::mex-6 vs. Ant:GFP::mex-5	-0.4389	-0.7391 to -0.1387	0.0012
Ant:GFP::mex-6 vs. Post:GFP::mex-6	-0.5088	-0.7798 to -0.2379	<0.0001
Ant:GFP::mex-6 vs. Post:GFP::mex-5	-1.132	-1.429 to -0.8340	<0.0001
Ant:GFP::mex-5 vs. Post:GFP::mex-6	-0.06998	-0.3552 to 0.2153	0.9196
Ant:GFP::mex-5 vs. Post:GFP::mex-5	-0.6928	-1.004 to -0.3821	<0.0001
Post:GFP::mex-6 vs. Post:GFP::mex-5	-0.6228	-0.9054 to -0.3403	<0.0001
**Figure S1C**	*GFP::mex-6* vs *GFP::mex-6(S403A)*		
**Stage**	**P**	**DF**	**t**
Steady state	<0.000001	46	12.11
**Figure S1D**	*GFP::mex-6* vs *GFP::mex-6(S403A)*		
	**F (DFn, DFd)**	**P**	
Interaction	F (1, 117) = 14.41	0.0002	

Row factor	F (1, 117) = 19.44	<0.0001	
Column factor	F (1, 117) = 23.21	<0.0001	
**Tukey's test**	**Predicted (LS) mean diff.**	**95% CI of diff.**	**Adjusted P**
Ant:GFP::mex-6 vs. Ant:GFP::mex-6(S403A)	0.06334	-0.1586 to 0.2853	0.879
Ant:GFP::mex-6 vs. Post:GFP::mex-6	-0.5088	-0.6873 to -0.3303	<0.0001
Ant:GFP::mex-6 vs. Post:GFP::mex-6(S403A)	0.02534	-0.2177 to 0.2684	0.9929
Ant:GFP::mex-6(S403A) vs. Post:GFP::mex-6	-0.5722	-0.7854 to -0.3590	<0.0001
Ant:GFP::mex-6(S403A) vs. Post:GFP::mex-6(S403A)	-0.038	-0.3075 to 0.2315	0.983
Post:GFP::mex-6 vs. Post:GFP::mex-6(S403A)	0.5342	0.2991 to 0.7693	<0.0001
**Figure S1E**	*GFP::mex-6* vs *mNG::mex-6*		
	**F (DFn, DFd)**	**P**	
Interaction	F (1, 159) = 6.061	P=0.0149	
Row factor	F (1, 159) = 71.35	P<0.0001	
Column factor	F (1, 159) = 1.477	P=0.2260	
**Tukey's test**	**Predicted (LS) mean diff.**	**95% CI of diff.**	**Adjusted P**
Ant:GFP::mex-6 vs. Ant:mNG::mex-6	-0.05814	-0.2338 to 0.1175	0.8256
Ant:GFP::mex-6 vs. Post:GFP::mex-6	-0.5088	-0.6781 to -0.3396	<0.0001
Ant:GFP::mex-6 vs. Post:mNG::mex-6	-0.3373	-0.5140 to -0.1606	<0.0001
Ant:mNG::mex-6 vs. Post:GFP::mex-6	-0.4507	-0.6163 to -0.2851	<0.0001
Ant:mNG::mex-6 vs. Post:mNG::mex-6	-0.2792	-0.4525 to -0.1059	0.0003
Post:GFP::mex-6 vs. Post:mNG::mex-6	0.1715	0.004734 to 0.3383	0.0413
**Figure S3A**	*GFP::mex-5; ctrl(RNAi)* vs *GFP::mex-5; mex-6(RNAi)*, per stage		
**Stage**	**P**	**DF**	**t**
Early embryos	0.831	23	0.216
Relaxation at posterior	0.178	33	1.376
Ruffling	0.032	39	2.221
Pseudo-cleavage	0.0003	43	3.961
Pronuclear meeting	0.0003	42	3.903
Pronuclear centration	<0.000001	42	5.819
Steady state	<0.000001	48	7.211
**Figure S3B**	*GFP::mex-6; ctrl(RNAi)* vs *GFP::mex-6; mex-5(RNAi)*, per stage		
**Stage**	**P**	**DF**	**t**
Relaxation at posterior	0.443	21	0.782
Ruffling	0.034	22	2.267
Pseudo-cleavage	0.0005	28	3.922
Pronuclear meeting	0.0005	29	3.906
Pronuclear centration	0.0088	30	2.802

Steady state	0.013	28	2.638
**Figure S4A**	*mNG::mex-5* vs *mNG::mex-5(T186A)*		
**Stage**	**P**	**Sum of ranks**	**Mann-Whitney U**
Steady state	0.015	732, 1969	258
**Figure S4A**	*mNG::mex-6* vs *mNG::mex-6(T190A)*		
**Stage**	**P**	**Sum of ranks**	**Mann-Whitney U**
Steady state	0.018	2087, 2284	744
**Figure S4B**	*mNG::mex-5* vs *mNG::mex-5(T186A)*		
	**F (DFn, DFd)**	**P**	
Interaction	F (1, 60) = 0.6658	0.4178	
Row factor	F (1, 60) = 50.19	<0.0001	
Column factor	F (1, 60) = 0.8299	0.366	
**Tukey's test**	**Predicted (LS) mean diff.**	**95% CI of diff.**	**Adjusted P**
Ant:mNG::mex-5 vs. Ant:mNG::mex-5(T186A)	-0.009216	-0.3716 to 0.3532	0.9999
Ant:mNG::mex-5 vs. Post:mNG::mex-5	-0.6078	-0.9468 to -0.2689	<0.0001
Ant:mNG::mex-5 vs. Post:mNG::mex-5(T186A)	-0.7753	-1.138 to -0.4129	<0.0001
Ant:mNG::mex-5(T186A) vs. Post:mNG::mex-5	-0.5986	-0.9610 to -0.2362	0.0003
Ant:mNG::mex-5(T186A) vs. Post:mNG::mex-5(T186A)	-0.7661	-1.150 to -0.3817	<0.0001
Post:mNG::mex-5 vs. Post:mNG::mex-5(T186A)	-0.1675	-0.5298 to 0.1949	0.616
**Figure S4C**	*mNG::mex-6* vs *mNG::mex-6(T190A)*		
	**F (DFn, DFd)**	**P**	
Interaction	F (1, 135) = 0.5078	0.4773	
Row factor	F (1, 135) = 56.25	<0.0001	
Column factor	F (1, 135) = 7.080	0.0087	
**Tukey's test**	**Predicted (LS) mean diff.**	**95% CI of diff.**	**Adjusted P**
Ant:mNG::mex-6 vs. Ant:mNG::mex-6(T190A)	0.06623	-0.05849 to 0.1909	0.5132
Ant:mNG::mex-6 vs. Post:mNG::mex-6	-0.2792	-0.3954 to -0.1630	<0.0001
Ant:mNG::mex-6 vs. Post:mNG::mex-6(T190A)	-0.1645	-0.2892 to -0.03978	0.0044
Ant:mNG::mex-6(T190A) vs. Post:mNG::mex-6	-0.3454	-0.4708 to -0.2200	<0.0001
Ant:mNG::mex-6(T190A) vs. Post:mNG::mex-6(T190A)	-0.2307	-0.3641 to -0.09740	<0.0001
Post:mNG::mex-6 vs. Post:mNG::mex-6(T190A)	0.1147	-0.01072 to 0.2401	0.0861

Supplementary Movies

Movie S1 (separate file). MEX-6 and MEX-5 gradient establishment in the one-cell *C. elegans* embryos. Time-lapse movies of the first division of one-cell embryos of the *mNG::mex-6* (A) and of the *mNG::mex-5* (B) strains.

Movie S2 (separate file). Degradation patterns of the MEX-5 and MEX-6 proteins are different and depend on the proteins' ZF domains. Time-lapse movies of the cell divisions from the 4-cell stage embryos of the *mNG::mex-5* (A) and of the *mNG::mex-6* (B) strains reveal different degradation rates for the two proteins, with MEX-5 degraded in somatic cells at late 4-cell stage. In the chimera where MEX-5 ZF domains were replaced with those of MEX-6 (*mNG::mex-5(MEX-6 ZF)*) (C), MEX-5(MEX-6 ZF) levels remained high in the soma cells, with an homogeneous distribution in the embryo similar to wild-type MEX-6. Vice versa, in the chimera *mNG::mex-6(MEX-5 ZF)* (D), MEX-6(MEX-5 ZF) is degraded in the somatic cells similarly to wild-type MEX-5.

SI References

1. S. Brenner, The genetics of Caenorhabditis elegans. *Genetics* **77**, 71-94 (1974).
2. C. M. Schubert, R. Lin, C. J. de Vries, R. H. Plasterk, J. R. Priess, MEX-5 and MEX-6 function to establish soma/germline asymmetry in early C. elegans embryos. *Mol Cell* **5**, 671-682 (2000).
3. S. Barbieri, A. Nurni Ravi, E. E. Griffin, M. Gotta, Modeling protein dynamics in Caenorhabditis elegans embryos reveals that the PLK-1 gradient relies on weakly coupled reaction-diffusion mechanisms. *Proc Natl Acad Sci U S A* **119**, e2114205119 (2022).
4. J. Schindelin *et al.*, Fiji: an open-source platform for biological-image analysis. *Nat Methods* **9**, 676-682 (2012).
5. M. Kang, C. A. Day, A. K. Kenworthy, E. DiBenedetto, Simplified equation to extract diffusion coefficients from confocal FRAP data. *Traffic* **13**, 1589-1600 (2012).
6. A. Noatynska, C. Panbianco, M. Gotta, SPAT-1/Bora acts with Polo-like kinase 1 to regulate PAR polarity and cell cycle progression. *Development* **137**, 3315-3325 (2010).
7. P. Bankhead *et al.*, QuPath: Open source software for digital pathology image analysis. *Sci Rep* **7**, 16878 (2017).
8. M. Pachitariu, C. Stringer, Cellpose 2.0: how to train your own model. *Nat Methods* **19**, 1634-1641 (2022).
9. N. N. Huang, C. P. Hunter, The RNA binding protein MEX-3 retains asymmetric activity in the early Caenorhabditis elegans embryo in the absence of asymmetric protein localization. *Gene* **554**, 160-173 (2015).
10. E. E. Griffin, D. J. Odde, G. Seydoux, Regulation of the MEX-5 gradient by a spatially segregated kinase/phosphatase cycle. *Cell* **146**, 955-968 (2011).
11. D. J. Dickinson, F. Schwager, L. Pintard, M. Gotta, B. Goldstein, A Single-Cell Biochemistry Approach Reveals PAR Complex Dynamics during Cell Polarization. *Dev Cell* **42**, 416-434.e411 (2017).
